# Leveraging multimodal cancer immunotherapy to amplify the efficacy of oncolytic viruses

**DOI:** 10.1186/s40164-026-00805-0

**Published:** 2026-07-02

**Authors:** Qiying Cai, Louqian Zhang, Lingkai Kong, Juan Fang, Juan Xu, Xiaosong Gu, Wujun Li, Chunping Jiang, Junhua Wu

**Affiliations:** 1 State Key Laboratory of Pharmaceutical Biotechnology, Center of Medical Big Data, Division of Hepatobiliary and Transplantation Surgery, Department of General Surgery, Nanjing Drum Tower Hospital, the Affiliated Hospital of Medical School, Suqian Scientific Research Institute of Nanjing University Medical School, Medical School, Nanjing University, Nanjing, China; 2 Jinan Microecological Biomedicine Shandong Laboratory, Building 1, Jinan Medical and Health Science and Technology Innovation Industrial Park, No. 288, Jiqi Road, Huayin District, Jinan, Shandong China; 3 “Nanjing University-Gulou” Joint Laboratory of AI and Healthcare BigData, National Institute of Healthcare Data Science at Nanjing University, School of Life Sciences, Jiangsu Key Laboratory of Molecular Medicine, Nanjing University, Nanjing, 210093 China; 4 Department of Hepatobiliary and Pancreatic Surgery, The Second Affiliated Hospital of Fujian Medical University, Quanzhou, 362000 Fujian China; 5 Renhuai People’s Hospital, Renhuai, 564055 Guizhou China

**Keywords:** Oncolytic virus, Cancer immunotherapy, Combination therapy, Immune checkpoint inhibitors, Cell therapy, Cancer vaccine, Immunity, Tumor microenvironment, Artificial intelligence, Gut microbiota

## Abstract

Oncolytic viruses (OVs) represent a versatile platform for cancer immunotherapy, capable of selectively infecting and lysing tumor cells while triggering systemic antitumor immunity. However, their therapeutic efficacy remains limited by antiviral immunity, restricted intratumoral spread, and an immunosuppressive tumor microenvironment. This review highlights emerging strategies to potentiate OV efficacy through rational combination with complementary immunotherapies, including immune checkpoint inhibitors, adoptive cell therapies, cancer vaccines, and small-molecule immunomodulators. These synergistic interventions can remodel the tumor microenvironment, enhance immune cell infiltration and activation, reverse immunosuppressive feedback, promote immunogenic cell death, normalize the tumor vasculature, and modulate the gut microbiota, collectively amplifying OV replication and oncolytic potency. The integration of artificial intelligence and multiomics profiling further enables precise patient stratification and optimization of combination regimens. In parallel, advanced engineering strategies, such as arming OVs with immunomodulatory transgenes and mitigating host antiviral responses, further reinforce these effects. Together, these approaches illustrate how multimodal immunotherapy can overcome the intrinsic limitations of OVs, enabling durable antitumor immunity. This review underscores the central role of combination strategies in OV-based immuno-oncology and outlines future directions to accelerate their clinical translation.

## Introduction

The notion that microbial infections could influence tumor progression emerged in the early 19th century, when spontaneous tumor regression was documented in cancer patients following acute bacterial infections [1]. These clinical observations spurred investigative efforts into the use of microorganisms as antitumor agents [2]. By the mid-19th century, viral infections had also been reported to confer incidental antitumor benefits, laying the conceptual groundwork for the development of oncolytic virus (OV)-based cancer immunotherapy [3]. OVs are characterized by their ability to selectively infect and lyse malignant cells while sparing normal tissues [4]. These viruses are broadly categorized into DNA and RNA viruses (Table 1) [5]. DNA viruses, such as adenovirus (Adv) [6], vaccinia virus (VV) [7], and herpes simplex virus (HSV) [8], typically possess larger genomes, exhibit greater genetic stability, and support robust viral replication [9–11]. In contrast, RNA viruses, including reovirus (RV) [12], measles virus (MV) [13], Newcastle disease virus (NDV) [14], and vesicular stomatitis virus (VSV) [15], leverage the host translational machinery for efficient gene expression and rapidly stimulate innate immune responses [16].

**Table 1:** Oncolytic viruses and their properties for tumor immunotherapy

Virus	Genome	Size	Virion	Access mechanism	Site of replication	Strategy of attenuation
Adv	dsDNA	28–42 kbp	Naked	CAR, CD46 [347]	Nucleus	Deletion of E1/E3 regions
HSV	dsDNA	125–240 kbp	Enveloped	HVEM, Nectin-1, Nectin-2 [348]	Nucleus	Deletion of ICP34.5, ICP47, ICP6, UL23 or UL40
VV	dsDNA	130–375 kbp	Enveloped	Endocytosis [349]	Cytoplasm	Deletion of TK or Vaccinia Growth Factor
CVA	ssRNA(+)	7.2–7.4 kb	Naked	CAR, ICAM-1, DAF [350, 351]	Cytoplasm	None
NDV	ssRNA(−)	15.1–15.9 kb	Enveloped	Sialic acid [352]	Cytoplasm	Deletion of V Protein
MV	ssRNA(−)	15.9 kb	Enveloped	SLAM, Nectin-4, CD46 [353, 354]	Cytoplasm	None
VSV	ssRNA(−)	11.2 kb	Enveloped	Low-density lipoprotein receptor [355]	Cytoplasm	Modified matrix protein
RV	dsRNA	23.5 kb	Naked	Junctional adhesion molecule A [356]	Cytoplasm	None
SVV	ssRNA(+)	7.3 kb	Naked	Endocytosis [357]	Cytoplasm	Modification of capsid proteins
Alphavirus	ssRNA(+)	12 kb	Enveloped	Matrix remodeling associated 8 [358]	Cytoplasm	None

Adv, adenovirus; HSV, herpes simplex virus; VV, vaccinia virus; CVA, coxsackievirus A; NDV, Newcastle disease virus; MV, measles virus; VSV, vesicular stomatitis virus; RV, reovirus; SVV, Seneca Valley virus; CAR, coxsackievirus and adenovirus receptor; HVEM, herpesvirus entry mediator; ICP, infected cell protein; UL, unique long region; TK, thymidine kinase; ICAM-1, intercellular cell adhesion molecule-1; DAF, decay accelerating factor; SLAM, signaling lymphocytic activation molecule

The first oncolytic virotherapy approved was Rigvir, although it is not genetically engineered [17]. Since then, three genetically engineered OVs have attained clinical approval worldwide: H101 [18], talimogene laherparepvec (T-VEC) [19], and G47∆ [20]. Through rational genetic modification, contemporary OVs achieve enhanced tumor specificity, reduced off-target toxicity [21], and improved immunostimulatory capacity. These engineered viruses modulate the tumor microenvironment (TME) [22], provoke immunogenic cell death (ICD) [23, 24], and promote antitumor immunity through direct oncolysis [25], systemic immune activation [26], and enhanced immune cell infiltration [27], thereby emerging as promising platforms for personalized cancer therapy [28].

Recent advances in artificial intelligence (AI) and bioinformatics are further accelerating the development of precision OV therapeutics. AI-driven analysis of multiomics data (e.g., genomics and transcriptomics) enables the prediction of optimal OV strains and genetic modifications tailored to individual patients [29]. Moreover, AI facilitates the characterization of TME features to guide viral delivery strategies and identify synergistic drug–OV combinations via computational repurposing approaches. These innovations hold significant potential for enhancing the precision, safety, and clinical efficacy of OV regimens.

**Fig. 1:**
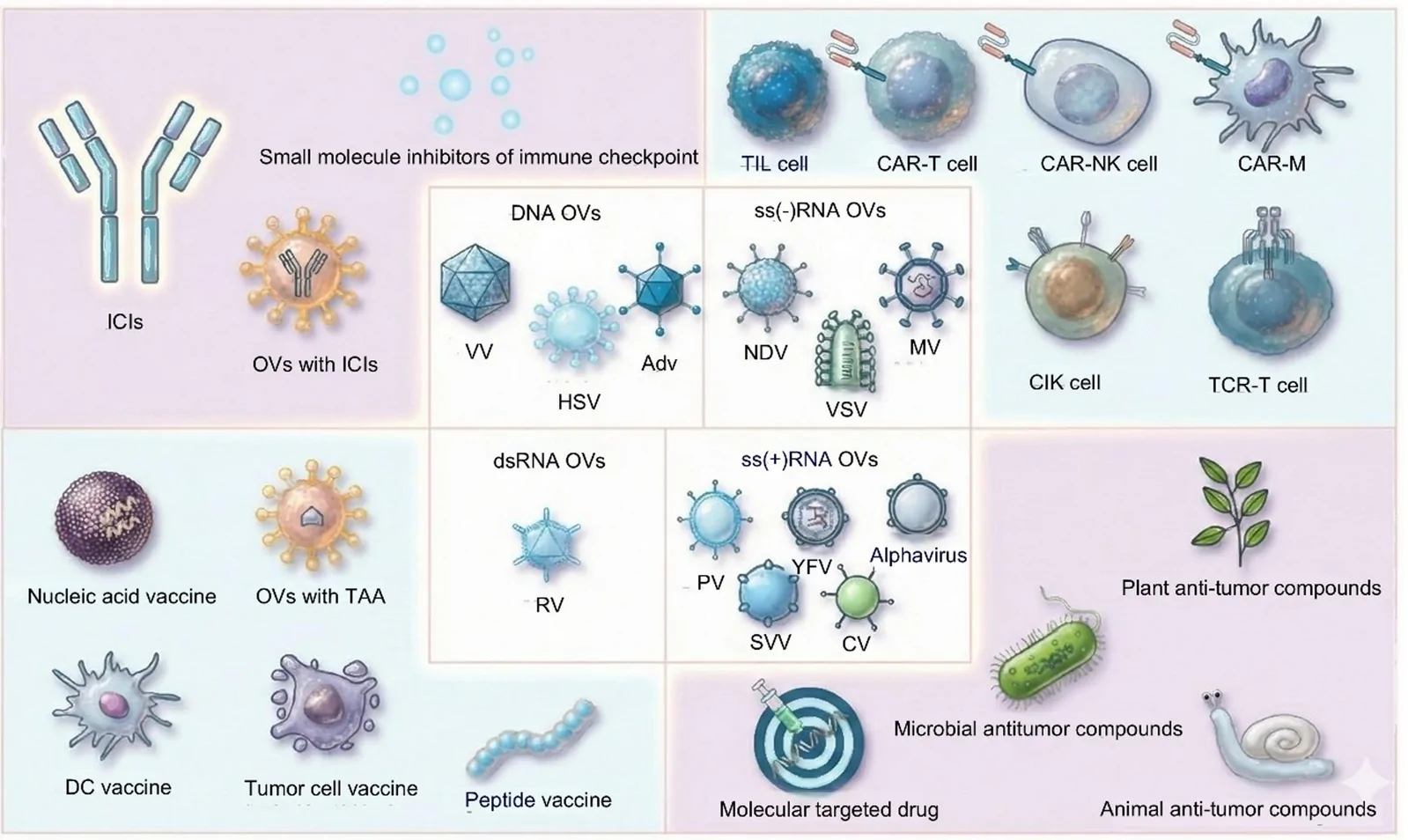
Combination strategies for OV therapy. OVs are integrated with diverse therapeutic modalities to enhance antitumor efficacy. Depending on the viral backbone, OVs include DNA viruses (VV, HSV, Adv), dsRNA viruses (RV), ss(−)RNA viruses (NDV, MV, VSV), and ss(+)RNA viruses (PV, YFV, SVV, CV, Alphavirus). OVs can be combined with ICIs or small-molecule inhibitors to potentiate immune activation; with adoptive cell therapies such as TILs, CAR-T cells, TCR-T cells, CAR-NK cells, CIK cells, and CAR-M cells to enhance cytotoxicity; and with various vaccine strategies, including nucleic acid, peptide, dendritic cell, and tumor cell vaccines, to strengthen antigen presentation. Additional synergistic approaches include integration with molecular targeted drugs, plant- or microbial-derived antitumor compounds, and animal-derived therapeutic agents. PV: poliovirus, YFV: yellow fever virus, SVV: Seneca Valley virus, CV: coxsackievirus, ICIs: immune checkpoint inhibitors, TIL: tumor-infiltrating lymphocyte, CAR: chimeric antigen receptor, NK cell: natural killer cell, M: macrophage, CIK: cytokine-induced killer, TCR: T-cell receptor-engineered, DC: dendritic cell

In this review, we explore how OVs amplify multimodal immunotherapy. We innovatively integrate AI-assisted OV design, vascular normalization, and microbiota–immune axis modulation into a unified framework and systematically elucidate the synergistic mechanisms between OVs and four major immunotherapeutic modalities (Fig. 1). Our objective is to provide a comprehensive foundation for the use of OVs as central components of next-generation cancer immunotherapy.

## Oncolytic mechanisms

Having outlined the historical development and engineering strategies of OVs, attention is now turned to the mechanistic basis of their antitumor effects. The following section explores the mechanisms by which OVs selectively lyse tumor cells and initiate immune activation within the TME.

### Immune activation: from in situ oncolysis to a systemic antitumor response

**Fig. 2:**
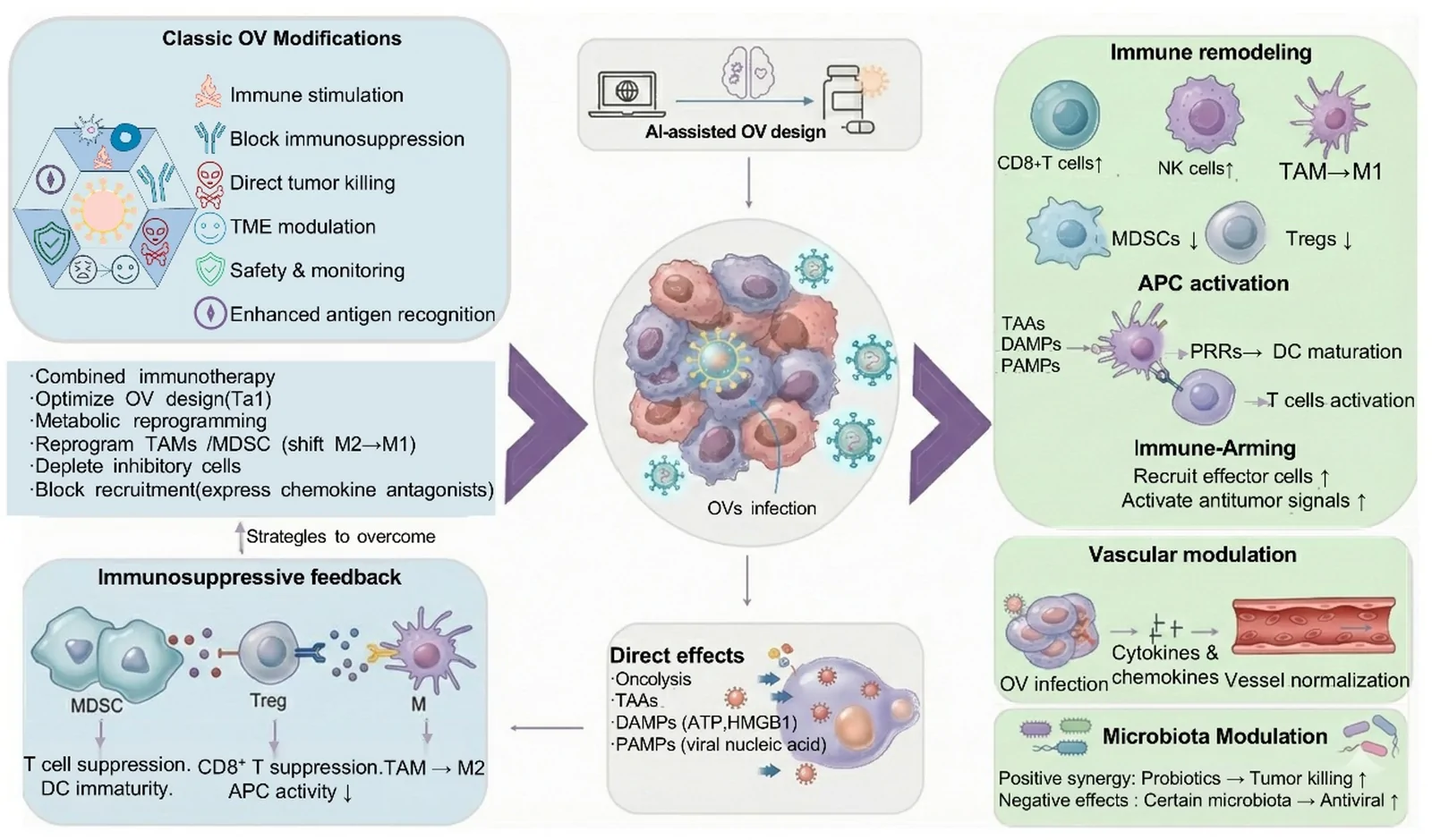
Mechanistic landscape of OV immunotherapy. OVs exert antitumor effects through both direct oncolysis and immune activation within the tumor microenvironment (TME). Classical OV engineering strategies enhance immune stimulation, alleviate immunosuppression, and improve tumor selectivity. AI-assisted OV design enables rational viral engineering and multimodal combination strategies to overcome immunosuppressive feedback mechanisms, including the accumulation of myeloid-derived suppressor cells (MDSCs) and regulatory T cells (Tregs). Upon infection, OVs induce immunogenic cell death (ICD) and trigger the release of damage-associated molecular patterns (DAMPs) and pathogen-associated molecular patterns (PAMPs), leading to dendritic cell maturation, T-cell priming, and immune landscape remodeling. Additional mechanisms include vascular normalization, macrophage repolarization, and microbiota modulation, collectively promoting durable antitumor immunity. ROS, reactive oxygen species; NO, nitric oxide; PRRs, pattern recognition receptors

The antitumor efficacy of OVs stems from a dual mechanism: direct tumor cell lysis and the subsequent initiation of a robust, systemic antitumor immune response (Fig. 2). The foundational event is the selective replication of OVs within tumor cells, exploiting their frequently dysregulated antiviral signaling pathways. This replication leads to ICD, a process characterized by the release of tumor-associated antigens (TAAs), damage-associated molecular patterns (DAMPs), and viral pathogen-associated molecular patterns (PAMPs) [30, 31]. These signals collectively function as potent in situ danger signals, attracting and activating antigen-presenting cells (APCs), such as dendritic cells (DCs), to the TME [32].

The engineered oncolytic HSV T-VEC exemplifies this paradigm. Key indicators of ICD following T-VEC treatment include surface exposure of calreticulin (an ‘eat-me’ signal), release of ATP (a chemotactic signal for immune cells), and upregulation of co-stimulatory molecules on DCs [33]. This localized immunogenic cascade culminates in the priming and activation of tumor-specific cytotoxic T lymphocytes (CTLs), which mediate the destruction of infected and uninfected tumor cells alike, a phenomenon known as antigen spreading. Critically, this response can overcome local immunosuppression. Clinical evidence from stage IIIc/IV melanoma patients demonstrates that intratumoral T-VEC injection induces potent local and systemic antigen-specific T-cell responses, concurrently reducing levels of immunosuppressive cell populations, including regulatory T cells (Tregs) and myeloid-derived suppressor cells (MDSCs) [34–36]. In a phase II clinical trial conducted by Todo et al., patients with glioblastoma were treated with intratumoral injections of the oncolytic HSV G47Δ. Following treatment, patients exhibited a sustained increase in the number of tumor-infiltrating CD4⁺/CD8⁺ T cells, along with low levels of Foxp3⁺ Tregs. Notably, this adaptive immune response demonstrated long-term persistence; in patients who survived more than three years, the effector T cell infiltration status in the immune microenvironment remained detectable for over 50 months. These findings indicate that G47Δ not only kills tumor cells through direct oncolytic effects but also remodels the TME, inducing durable and highly specific antitumor immune memory [37]. Moreover, the immunomodulatory capacity of OVs extends beyond direct tumor infection. As demonstrated by Rajwani et al., infection of non-cancer cells with VSV^∆M51^ alone suffices to trigger tumor regression. This systemic effect is partially mediated by cytokine-driven DC activation and enhanced antigen cross-presentation, ultimately amplifying antitumor CD8⁺ T cell responses [38]. As a result, by transforming the immunologically ‘cold’ TME into a ‘hot’ inflamed state, OVs bridge innate and adaptive immunity to establish durable, systemic antitumor immunity.

### Armoring and targeted delivery strategies to evade neutralizing antibodies

A significant challenge in oncolytic virotherapy is the presence of preexisting virus-neutralizing antibodies due to prior exposure to wild-type viruses, which can potentially inhibit the therapeutic efficacy of systemically administered OVs [39, 40]. Notably, some clinical evidence suggests that neutralizing antibodies may not entirely abrogate antitumor activity and could even be associated with enhanced safety and treatment response in certain contexts [41, 42]. For instance, the oncolytic herpesvirus CAN-3110 was associated with improved survival and tumor clearance in a clinical trial (NCT03152318), with heightened antitumor immune responses observed particularly in seropositive patients [43]. Nevertheless, to maximize the potential of OVs, numerous innovative strategies have been developed to circumvent neutralization [44].

Current armoring approaches can be broadly categorized into three paradigms: (1) genetic modification of OVs to enhance tumor selectivity and reduce immunogenicity [45, 46]; (2) the use of cellular carriers, such as stem cells or immune cells, for precise tumor-targeted delivery and shielded transport [47]; and (3) physicochemical camouflage using biomaterials including polymers [48], hydrogels [49], synthetic lipid layers [50], or cell-derived membranes [51, 52]. These coatings mask viral surface epitopes, thereby minimizing recognition and clearance by neutralizing antibodies while improving circulatory stability and biodistribution [53]. Crucially, such modifications can concurrently enhance tumor cell entry efficiency; for example, liposome-encapsulated Adv demonstrates a ~ 100-fold increase in cytoplasmic delivery while evading antibody-mediated neutralization [54]. Similarly, Chen et al. developed a folate receptor-targeted graphene oxide-based nanocarrier (PEI-GOS-PEG-FA) to encapsulate oncolytic MV (MV-Edm), effectively shielding it from immune recognition and enabling efficient tumor-specific delivery even in the presence of anti-MV antibodies [55].

Cellular carriers, especially mesenchymal and neural stem cells, leverage innate tumor-tropic properties for efficient OV delivery [56, 57]. This approach has demonstrated promising clinical outcomes. In a phase I trial for patients with high-grade glioma, neural stem cell-delivered oncolytic Adv (NSC-CRAd-S-pk7) yielded a median progression-free survival of 9.05 months and an overall survival of 18.4 months (NCT03072134). Immunological analyses confirmed that high-dose treatment robustly enhanced antitumor immunity, characterized by increased CD8 + T cell infiltration and a more favorable cytokine profile [58, 59]. These strategies collectively highlight the evolving frontier of OV engineering aimed at overcoming immunological barriers to enhance therapeutic efficacy.

### Genetic armament of OVs to remodel the TME

Beyond their direct oncolytic activity that alters the TME, OVs can be genetically armed with a diverse array of therapeutic transgenes to actively promote immune cell infiltration and activation. These exogenous genes, including immunostimulatory factors [60], tumor-specific antigens [61, 62], noncoding RNAs [63], bispecific T-cell engagers [64], and chemokines [65], collectively enhance the immunomodulatory capacity of OVs and strengthen antitumor immunity by improving immune-mediated tumor recognition and destruction [5]. A summary of armed OVs under clinical investigation is provided in Table 2.

**Table 2:** Oncolytic viruses carrying exogenous genes identified in clinical studies

Virus	Biological agent	Exogenous gene	Cancer	Clinical trial	Clinical trial No
Adv	Ad-TD-nsIL12	IL12	Primary Pediatric Diffuse Intrinsic Pontine Glioma	I	NCT05717712
	DNX-2440	OX40L	Recurrent Glioblastoma	I	NCT03714334
	ADV/HSV-tk	HSV-TK	Recurrent High-Grade Glioma	II	NCT00870181
	TILT-123	TNFα, IL2	Solid Tumor	I	NCT04695327
	MEM-288	CD40L, IFNβ	Solid Tumors, NSCLC	I	NCT05076760
	Ad5-yCD/mutTKSR39rep-ADP	CD, HSV-TK, IL-12	Pancreatic Cancer	II	NCT04739046
	YSCH-01	L-IFN	Solid Tumor	I	NCT05180851
	NG-350 A	anti-CD40 antibody	Advanced or Metastatic Epithelial Tumors	I	NCT03852511
	BioTTT001	IL12	Peritoneal Metastases from Gastric Cancer	II	NCT06283121
	ONCOS-102	GMCSF	Metastatic or Unresectable Malignant Melanoma	II	NCT05561491
	VCN-01	RGDK, PH20	Metastatic Pancreatic Cancer	II	NCT05673811
	CG0070	GM-CSF	Bladder Cancer	II/III	NCT01438112
	TS-2021	IL-15	Recurrent Malignant Glioma	I	NCT06585527
	LOAd703	TMZ-CD40L, BBL	Pancreatic Cancer	I/II	NCT02705196
	NG-641	FAP-TAc, CXCL9, CXCL10, IFNa	Metastatic or Advanced Epithelial Tumors	I	NCT04053283
	NV-A01	ApoA1	Advanced Glioblastoma	I	NCT06193538
	KD01	tBID apoptosis protein	Cervical Malignancies	I	NCT06552598
	SynOV1.1	Gal4VP16	Locally Advanced or Metastatic Solid Tumors	I	NCT04612504
	Ad5 OBP-301 (Telomelysin)	hTERT	Advanced or Metastatic Gastroesophageal Adenocarcinoma	II	NCT03921021
HSV	T-VEC	GMCSF	Melanoma	III	NCT02263508
	oHSV2-PD-L1/CD3-BsAb	PD-L1/CD3-BsAb	Solid Tumors	I	NCT05938296
	OH2	GMCSF	Melanoma	III	NCT05868707
	T3011	IL-12 and PD-1 antibody	Melanoma	II	NCT05756556
	G207	LacZ	Recurrent/Progressive Pediatric High-Grade Gliomas	II	NCT04482933
	RP2	GALV-GP-R–, GM-CSF, anti-CTLA-4 antibody-like molecule	Metastatic Uveal Melanoma	II/III	NCT06581406
	RP3	4-1BB, GALV-GP-R–, anti-CTLA-4 antibody-like molecule	Advanced Unresectable or Metastatic Hepatocellular Carcinoma	II	NCT05733598
	ONCR-177	IL-12, FLT3LG ECD, CCL4, anti-PD-1 antibody, anti-CTLA-4 antibody	Solid Tumors	I	NCT04348916
	M032-HSV-1	IL-12	Recurrent Malignant Glioma	I/II	NCT05084430
	C134	IRS1	Recurrent Malignant Glioma	I	NCT06193174
	RP1	GALV-GP R-, GM-CSF	Advanced Cutaneous Squamous Cell Carcinoma	II	NCT04050436
	HF10	UL53, UL54	Melanoma	II	NCT03259425
VV	VV-GMCSF-Lact	GM-CSF, lactaptin	Recurrent/Refractory Metastatic Breast Cancer	I	NCT05376527
	hV01	IL-21	Advanced Solid Tumors	I	NCT05914376
	GC001	STRIP1, shRNA	Advanced Solid Tumors	I	NCT06508307
	GL-ONC1	Ruc-GFP, β-glucuronidase, β-galactosidase	Platinum-Resistant/Refractory Ovarian Cancer	III	NCT05281471
	Pexa-Vec	GM-CSF	Advanced Hepatocellular Carcinoma	III	NCT02562755
	CF33-hNIS-antiPDL1	NIS, Anti-PD-L1 antibody	Metastatic Triple Negative Breast Cancer	I	NCT05081492
	T601	FCU1	Advanced Malignant Solid Tumors	I/II	NCT04226066
	CF33-CD19	CD19	Advanced or Metastatic Solid Tumors	I	NCT06063317
	TG6002	FCU1	Advanced Gastro-intestinal Tumors	I/II	NCT03724071
	vvDD-hIL2	hIL2	Metastatic Gastrointestinal and Peritoneal Tumors	I	NCT07001592
	ASP9801	IL-7, IL-12	Advanced/Metastatic Solid Tumors	I	NCT03954067
	BT-001	Anti-CTLA-4 antibody, GM-CSF	Advanced/Metastatic Solid Tumors	I/II	NCT04725331
MV	MV-NIS	NIS	Ovarian, Fallopian, Peritoneal Cancer	II	NCT02364713
	TMV-018	SCD	Tumors of the Gastrointestinal Tract	I	NCT04195373
NDV	MEDI5395	GMCSF	Advanced Solid Tumors	I	NCT03889275
VSV	VSV-IFNβ-NIS	IFNβ, NIS	Solid Tumors	I/II	NCT03647163
	VSV-IFNβ/TYRP1	IFNβ, tyrosinase related protein 1	Melanoma	I	NCT03865212
Malabar	MG1MA3 (MG1 Maraba/MAGE-A3)	MAGE-A3	Incurable Advanced/Metastatic MAGE-A3-Expressing Solid Tumors	I/II	NCT02285816

Adv, adenoviruses; HSV, herpes simplex virus; VV, vaccinia virus; MV, measles virus; NDV, Newcastle disease virus; VSV, vesicular stomatitis virus; IL, interleukin; OX40L, Oxford 40 ligand; TK, thymidine kinase; TNFα, tumor necrosis factor α; CD40L, CD40 ligand; IFN, interferon; yCD, yeast cytosine deaminase; GM-CSF, granulocyte‒macrophage colony-stimulating factor; RGDK, an integrin-binding motif; PH20, hyaluronidase; TMZ-CD40L, trimerized membrane-bound CD40L; 4-1BBL, 4-1BB ligand; FAP-TAc, fibroblast activation protein-T-cell activator; CXCL, C-X-C motif chemokine ligand; ApoA1, apolipoprotein A1; tBID, truncated BH3 interacting domain death agonist; Gal4VP16, gal-4 DNA-binding domain-VP16 transactivation domain; hTERT, human telomerase reverse transcriptase; PD-L1, programmed death ligand 1; BsAb, bispecific antibody; LacZ, β-galactosidase gene; GALV-GP-R–, glycoprotein of gibbon ape leukemia virus; CTLA-4, cytotoxic T-lymphocyte-associated protein 4; FLT3LG ECD, FMS-like tyrosine kinase 3 ligand extracellular domain; CCL4, chemokine (C-C motif) ligand 4; IRS1, protein kinase R-evasion gene 1; UL, unique long region; STRIP1, Striatin-Interacting Protein 1; shRNA, short hairpin ribonucleic acid; Ruc-GFP, renilla luciferase-green fluorescent protein; NIS, sodium-iodide symporter; FCU1, FCU1 encodes a bifunctional fusion protein combining cytosine deaminase and uracil phosphoribosyltransferase activity; SCD, stearoyl-coA desaturase; MAGE-A3, melanoma-associated antigen A3; NSCLC, non-small cell lung cancer

Among these, granulocyte‒macrophage colony‒stimulating factor (GM-CSF) remains one of the most widely applied and well-characterized transgenes [66]. OVs engineered to express GM-CSF enhance DC maturation and antigen presentation [67], and promote the polarization of macrophages toward an antitumor M1 phenotype [68]. ONCOS-102, an oncolytic Adv expressing GM-CSF, has advanced to phase II clinical trials [69]. In an earlier phase I study (NCT01598129), intratumoral administration of ONCOS-102 induced robust innate and adaptive immune activation and exhibited notable antitumor activity in patients with refractory solid tumors [70]. Remarkably, ONCOS-102 demonstrates potential to reverse resistance to anti-programmed death receptor 1 (PD-1) therapy. In patients with advanced melanoma refractory to PD-1 blockade, the combination of ONCOS-102 and pembrolizumab was well-tolerated and achieved an objective response rate (ORR) of 35%, with tumor regression observed in non-injected lesions in 53% of patients. Durable CD8 + T cell infiltration confirmed systemic immune activation, highlighting a promising combinatorial strategy [71].

To address the immunosuppressive complexity of the TME, more sophisticated armed OVs have been developed. NDV-GT, an engineered NDV expressing porcine α1,3-galactosyltransferase, catalyzes the expression of α-galactosyl (αGal) epitopes on infected tumor cells. These epitopes are recognized by pre-existing natural anti-αGal antibodies, triggering complement-dependent cytotoxicity and antibody-dependent cellular cytotoxicity (ADCC). This leads to the release of platelet-activating factor and subsequent tumor vascular thrombosis and ischemic necrosis. Concurrently, antibody binding promotes tumor antigen release, activates DCs, and facilitates the infiltration of CD4 + and CD8 + T cells into tumors, accompanied by increased production of interferon-γ (IFN-γ), tumor necrosis factor-α (TNF-α), granzyme B, and perforin. Furthermore, NDV-GT modulates immunosuppression by inhibiting the PI3K–Akt–IKK–NF-κB pathway and reducing populations of Tregs and MDSCs, thereby sustaining a durable antitumor immune response through combined oncolysis, vascular targeting, and immunostimulation [72].

Similarly, HSV-1 (anti-TRAIL), an oncolytic herpesvirus encoding a single-chain antibody against tumor necrosis factor-related apoptosis-inducing ligand (TRAIL), has shown efficacy in glioblastoma models. Intratumoral delivery mitigates TRAIL-mediated apoptosis of CD4 + and CD8 + T cells, a mechanism often exploited by astrocytes in the TME, while increasing T-cell infiltration. Transcriptomic analyses revealed that treatment enhances proinflammatory T-cell functions, promotes Th1-type responses, activates tumoricidal pathways in tumor-associated macrophages (TAMs), and strengthens TAM–T-cell crosstalk. This multifaceted remodeling of the immunosuppressive TME synergizes with direct viral oncolysis to potentiate antitumor immunity [73].

### Induction of durable antitumor immunity and immunological memory

The establishment of long-term protective immunity is a critical objective of oncolytic virotherapy, essential for preventing tumor immune escape and achieving sustained therapeutic efficacy [74]. VV, renowned for its historic role as the smallpox vaccine, is particularly notable for its ability to induce potent and durable immune protection [75, 76]. Recent research by Depeaux et al. further elucidated its oncolytic mechanism: VV preferentially infects terminally differentiated, hypoxic, and exhausted CD8⁺ T cells, as well as regulatory CD4⁺ T cells within tumors, resulting in viral replication and subsequent cell death. Notably, inhibiting T-cell apoptosis through Bcl2 overexpression markedly diminished tumor regression and survival, underscoring that VV-mediated elimination of immunosuppressive T cells is a central mechanism of its antitumor effect [77]. To enhance this immunogenic potential, engineered variants such as VV-α-TIGIT, a recombinant VV expressing a monoclonal antibody against T-cell immunoglobulin and the ITIM domain (TIGIT), have been developed. In murine ascites models, treatment with VV-α-TIGIT led to complete tumor regression in the majority of cases, accompanied by enhanced T-cell recruitment and activation within the TME. Furthermore, mice cured with VV-α-TIGIT resisted tumor rechallenge, demonstrating the induction of potent and long-lasting tumor-specific immunological memory [78].

B cells play an indispensable role in sustaining such antitumor immunity. As central mediators of humoral immunity [79], activated B cells produce tumor antigen-specific antibodies that facilitate opsonization, complement activation, and ADCC, thereby aiding in immune recognition and clearance of malignant cells [80]. Beyond antibody production, B cells engage with cellular immune components, including T cells, to orchestrate optimal antigen-specific memory responses and amplify overall antitumor immunity [81]. The essential role of B cells is exemplified by studies with OVH, an oncolytic HSV with deletions in ICP0 and ICP34.5. B-cell depletion experiments revealed a significant reduction in OVH-induced antitumor efficacy, confirming that B cells are crucial for achieving maximal therapeutic outcomes [82].

### Overcoming OV-induced immunosuppressive feedback in the TME

The interaction between OVs and the TME entails a complex duality. While OVs are renowned for their immunostimulatory properties, accumulating evidence indicates that they can also paradoxically reinforce immunosuppressive mechanisms within the TME [83, 84]. During the process of virus-mediated remodeling, OVs may inadvertently facilitate the recruitment and activation of immunosuppressive cell populations. For instance, cytokine cascades initiated by OV infection can recruit MDSCs and Tregs [85–87]. Similarly, DAMPs released from lysed tumor cells, though instrumental in activating innate immunity, can concomitantly trigger inhibitory signaling pathways [88]. Moreover, robust but dysregulated T-cell activation may lead to upregulation of exhaustion markers such as PD-1 and T-cell immunoglobulin and mucin domain-3 (TIM-3), further impairing antitumor immunity [89].

These immunosuppressive effects may arise as passive consequences of uncontrolled inflammatory responses or represent an active homeostatic feedback mechanism aimed at preventing immunopathological damage to normal tissues. Regardless of origin, this compensatory immunosuppression constitutes a major impediment to the efficacy of OV monotherapy, underscoring the necessity of combinatorial approaches and rational viral engineering to counteract these resistance mechanisms. Notable strategies are emerging to overcome these limitations. Liu et al. demonstrated that oncolytic Adv therapy induced M2 polarization of TAMs, which curtailed its antitumor effects. Co-administration of thymosin α1 (Tα1) effectively reprogrammed TAMs toward a proinflammatory M1 phenotype, reduced Treg accumulation, and enhanced CD8 + T cell cytotoxicity. These findings were further validated using an engineered Adv expressing Tα1 (Adv-Tα1), which consistently reversed immunosuppressive microenvironments and improved therapeutic outcomes [90]. In a separate study, Wang et al. reported that conventional Adv therapy not only increased the prevalence of M2-TAMs but also diminished the infiltration of effector memory and effector CD8 + T cells, thereby compromising durable immunity. To address this, the team developed two novel recombinant Advs, Adv-NE and Adv-PPE, encoding neutrophil elastase and porcine pancreatic elastase, respectively. These constructs induced pyroptotic cell death and facilitated the release of high-mobility group box 1 (HMGB1), which in turn activated the TLR4–MyD88–NF-κB–NLRP3 axis in macrophages, promoting M1 polarization and restoring CD8 + T cell infiltration. This approach significantly enhanced long-term antitumor immunity in colorectal cancer models [91].

Elucidating the precise mechanisms underlying OV-induced immunosuppressive feedback is therefore essential for developing more effective combination regimens, circumventing resistance, and ultimately improving clinical response rates and survival in cancer patients.

### Modulation of the tumor vasculature by OVs: from destruction to normalization

The targeted modulation of the tumor vasculature represents a promising frontier in oncolytic virotherapy, with strategies ranging from the targeted destruction of aberrant vessels to the active induction of vascular normalization [92, 93]. Tumor progression is accompanied by excessive and disorganized angiogenesis driven by hypoxia-induced factors such as vascular endothelial growth factor [94] and angiotensin [95]. This results in a dysfunctional vasculature characterized by high permeability, poor perfusion, and intensified hypoxia, which exacerbate immunosuppression, metastasis, and drug delivery barriers [96–101].

OVs can directly disrupt tumor-supporting blood vessels by infecting and lysing endothelial cells within the tumor vasculature, thereby compromising nutrient and oxygen supply and inhibiting tumor growth [102]. For example, intravenous administration of replication-competent VSV significantly reduces tumor vascular density through direct infection of endothelial cells and subsequent neutrophil-mediated vascular occlusion [103]. Similarly, the engineered VV-JX-594 selectively targets tumor endothelial cells via the RAS/MAPK pathway, inducing widespread thrombotic necrosis and demonstrating clinical efficacy against treatment-resistant hepatocellular carcinoma without damaging healthy tissues [104].

However, excessive vascular disruption may exert paradoxical effects on the TME. Vascular collapse can impair immune cell trafficking, limit drug delivery, and exacerbate hypoxia, thereby promoting immune exclusion and tumor invasiveness [105]. In this context, combination with ICIs is particularly important. OV-induced immunogenic cell death enhances antigen presentation, while ICIs restore T cell function and amplify antitumor immunity. In addition, OV-triggered inflammatory cytokines can increase vascular permeability and partially restore perfusion, facilitating immune infiltration and alleviating hypoxia-driven immunosuppression [106]. Thus, OV–ICI combinations help rebalance the TME and convert vascular disruption–associated liabilities into immunological advantages.

Beyond vascular destruction, a more refined approach involves OV-mediated vascular normalization. The inflammatory response triggered by OV infection can recruit immune cells into tumor cores and reprogram the expression of pro- and anti-angiogenic factors within the TME. This rebalancing promotes the maturation and stabilization of vascular structures, alleviates hypoxia, and improves immune cell infiltration and function. A notable example is Ad-IL-15, an interleukin (IL)-15-expressing oncolytic Adv, which operates through a dual mechanism: it activates the STING–TBK1–IRF3 pathway to enhance DC maturation and promote T/natural killer (NK) cell recruitment, while concurrently inducing vessel normalization and the formation of tertiary lymphoid structures. This coordinated immunovascular remodeling mitigates immunosuppressive barriers and significantly enhances antitumor immunity [107].

Thus, OVs offer a versatile toolkit for modulating the tumor vasculature and are capable of both disrupting pathological angiogenesis and promoting functional vascular recovery, thereby opening new avenues for combination therapy in solid tumors.

### The gut microbiota: a systemic regulator of oncolytic virotherapy efficacy

The gut microbiota is increasingly recognized as a pivotal systemic factor that shapes immune homeostasis and significantly influences the efficacy of oncolytic virotherapy by modulating vascular and immune remodeling within the TME [108, 109]. Through microbial metabolites and signaling via pattern recognition receptors, the gut microbiome can exert remote effects on immune cell function in distal tumors and directly impact the outcomes of OV treatment. Notably, the gut microbiota also critically regulates systemic type I interferon (IFN-I) signaling, thereby shaping host antiviral immunity. Microbiota-derived signals promote tonic IFN-I responses and prime dendritic cells and other innate immune cells, establishing a heightened antiviral state. In the context of oncolytic virotherapy, this microbiota–IFN axis represents a double-edged sword: while IFN-I enhances antitumor immunity by promoting DC maturation and cytotoxic lymphocyte activation, it can also restrict viral replication and intratumoral spread, potentially limiting OV efficacy [110].

For instance, the oncolytic VSVΔ51 has been shown to disrupt gut microbiota equilibrium and compromise intestinal barrier integrity, thereby attenuating antitumor immunity. Restoration of a healthy microbial community through supplementation with *Lactobacillus acidophilus* not only repairs the barrier, via upregulation of tight junction proteins such as Occludin and E-cadherin, but also augments cytotoxic CD8⁺ T cell responses in the TME, significantly enhancing the antihepatocellular carcinoma activity of VSVΔ51 [111]. Similarly, orally administered oncolytic RV (RC402) engages with the host immune system at Peyer’s patches in the terminal ileum. It promotes the expansion of IgA⁺ antibody-secreting cells in the lamina propria through MAdCAM-1⁺ vessels, thereby remodeling the gut microbiota. This process is microbiota-dependent and facilitates the activation of Batf3⁺ DCs, IFN I production, and priming of tumor-specific CD8⁺ T cells, ultimately leading to the infiltration of granzyme B⁺ T cells into distant tumors and the induction of apoptosis [12].

Emerging tools such as AI are further advancing this field by enabling detailed analysis of gut microbiome transcriptomic data. AI-driven approaches allow for the precise identification of microbial functional traits and pathways correlated with OV treatment response, supporting the rational design of personalized microbiome interventions. These may include the administration of specific probiotic strains, engineered bacteria, or the selection of suitable fecal microbiota transplantation donors [112]. The overarching goal of these strategies is to reprogram the composition and functional output of the gut microbiome, thereby potentiating systemic and intratumoral immune activation, improving OV therapeutic efficacy, and potentially increasing treatment tolerance.

### AI–driven optimization of oncolytic virotherapy

AI is emerging as a powerful enabler of oncolytic virotherapy, providing data-driven strategies to optimize viral design, predict therapeutic responses, and guide rational combination regimens. Computational approaches spanning machine learning, network-based modeling, and multi-omics integration are reshaping both the conceptual and translational landscape of OV research [113, 114].

Among these, machine learning constitutes a central component. Supervised models, including random forest, gradient boosting, and support vector machines, have been widely applied to transcriptomic and genomic datasets to predict tumor susceptibility to OV infection and therapeutic responsiveness [115, 116]. In parallel, unsupervised approaches such as hierarchical clustering and k-means enable patient stratification based on tumor immune phenotypes, including the distinction between immune-inflamed and immune-desert microenvironments [117]. More recently, deep learning architectures, including convolutional and transformer-based neural networks, have demonstrated utility in predicting regulatory element activity within viral genomes and extracting high-dimensional features from single-cell datasets [118, 119]. Notably, hybrid approaches integrating mechanistic mathematical modeling with machine learning further enhance predictive performance by incorporating features that capture tumor–virus–immune dynamics and pharmacokinetics, thereby improving tumor burden prediction and enabling more accurate, patient-specific modeling of therapeutic responses [120].

Beyond classical machine learning, integrative computational frameworks provide mechanistic insights into OV–host interactions. Network-based approaches, including protein–protein interaction and gene regulatory network analyses, identify key signaling hubs such as NF-κB and STING, thereby informing rational combination strategies [121]. Multi-omics integration methods, including matrix factorization, Bayesian models, and deep generative approaches, define OV-responsive molecular signatures and predictive biomarkers [122]. Concurrently, AI-driven frameworks integrating biophysical, pharmacokinetic, and immunological parameters improve the modeling of therapeutic responses, while hybrid approaches combining data-driven and mechanistic modeling enhance the characterization of tumor–virus–immune dynamics, enabling more accurate treatment optimization and patient stratification [123]. A recently developed AI-driven hybrid ecological framework integrating mechanistic tumor–virus–immune interaction modeling with genetic algorithms, differential evolution, and reinforcement learning achieved robust predictive performance in simulating OV treatment dynamics, with a mean squared error (MSE) below 0.02 and R² values exceeding 0.82. By incorporating viral replication, tumor growth kinetics, and immune-mediated responses into a unified computational platform, the model enabled accurate prediction of therapeutic outcomes and rational optimization of treatment strategies, highlighting the potential of AI-assisted multiscale modeling to advance precision oncolytic virotherapy [124]. Single-cell and spatial transcriptomic analyses further resolve OV-induced remodeling of the TME, including DC activation and T cell priming.

Advances in computational virology and synthetic biology are enabling the in silico optimization of viral constructs. Algorithms for codon usage optimization, promoter strength prediction, and genome-scale design support the engineering of OVs with enhanced tumor selectivity and controlled transgene expression, including immunomodulatory payloads [125]. Furthermore, AI-driven predictive models incorporating drug synergy metrics are increasingly used to identify optimal combination strategies between OVs and immunotherapies, such as ICIs and CAR-T cells [126]. Emerging reinforcement learning strategies further hold promise for adaptive treatment optimization [127]. Collectively, these AI-enabled approaches provide a comprehensive framework for integrating biological complexity into OV design and application, ultimately supporting the development of more precise, personalized, and effective oncolytic virotherapy strategies.

## Synergistic effects of OVs and ICIs

Immune checkpoints serve as critical regulators of immune homeostasis, functioning through finely tuned signaling pathways that maintain self-tolerance while modulating the intensity and duration of immune responses [128–131]. However, tumors frequently exploit these regulatory mechanisms by dysregulating immune checkpoint signaling, thereby evading immunosurveillance and destruction [132–134]. Key among these evasion strategies is the expression of checkpoint ligands, such as PD-L1, by tumor cells, which engage cognate receptors on immune cells (notably T cells) and transduce inhibitory signals that dampen antitumor immunity [135]. This ligand-receptor interplay effectively suppresses T-cell activation and effector functions, positioning immune checkpoints as central mediators of tumor immune escape. The development of ICIs represents a transformative advancement in cancer immunotherapy. By selectively blocking the interaction between checkpoint receptors and their ligands, ICIs release inhibitory brakes on immune bystander cells and rejuvenate antitumor immune responses [136]. To date, monoclonal antibodies targeting cytotoxic T lymphocyte-associated antigen-4 (CTLA-4) and the PD-1/PD-L1 axis have received clinical approval and are widely used in the treatment of malignancies such as melanoma, lymphoma, and non-small cell lung cancer [137, 138]. In parallel, a new generation of ICIs directed against emerging targets, including lymphocyte activation gene-3, TIM-3 [139], V-domain Ig suppressors of T-cell activation [140], glucocorticoid-induced tumor necrosis factor receptor [141], and B and T lymphocyte attenuators [142], are under active investigation to broaden the therapeutic landscape.

**Fig. 3:**
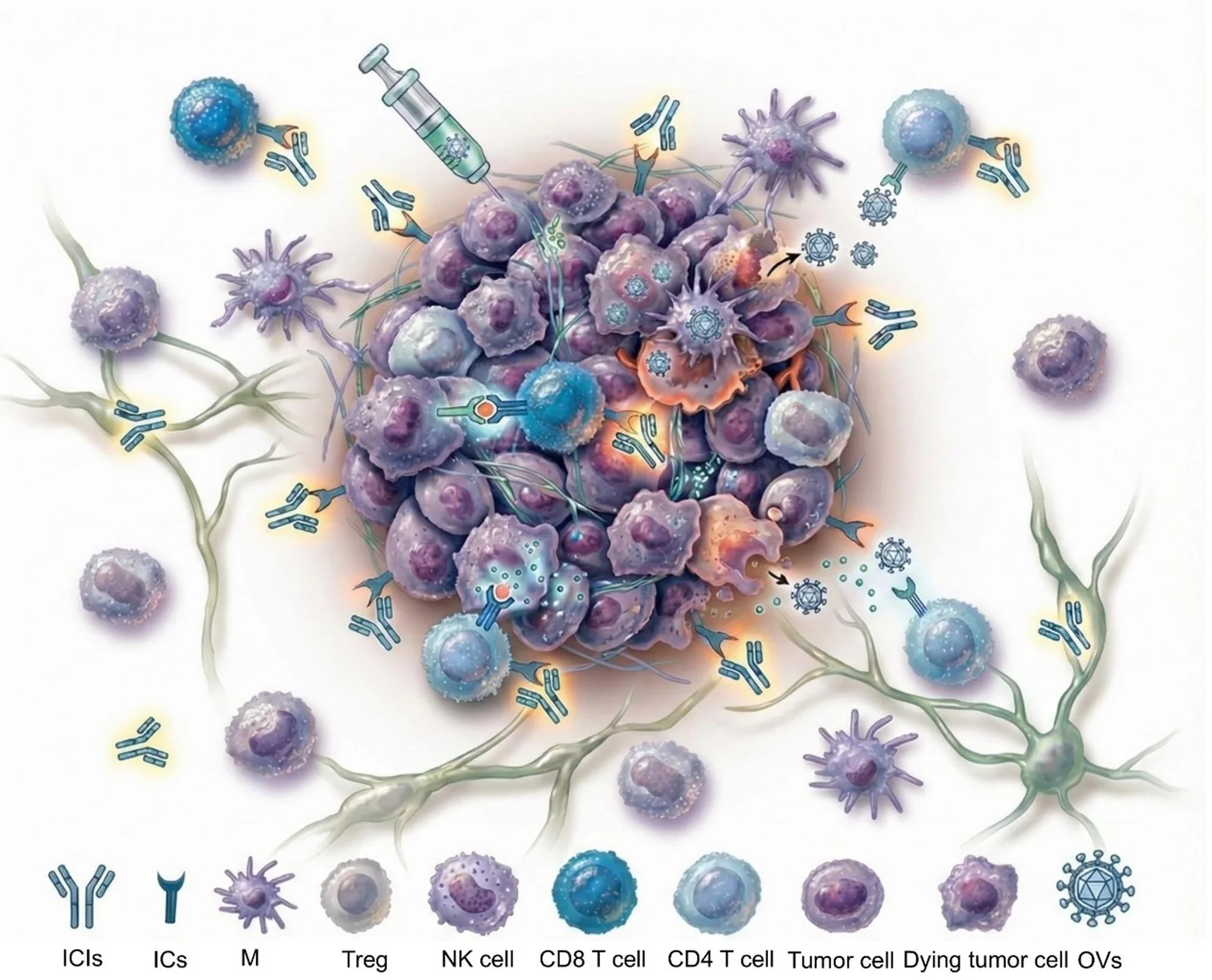
The combination of OVs and ICIs ameliorates the immunosuppressive TME. Schematic representation illustrating how the combination of OVs and ICIs remodels the immunosuppressive TME. Intratumoral administration of OVs selectively infects and lyses tumor cells, promoting immune cell infiltration and activation. The therapy enhances the recruitment of CD8⁺ T cells, CD4⁺ T cells, and NK cells, while alleviating Treg-mediated suppression and reprogramming macrophages toward a proinflammatory phenotype. ICIs further potentiate antitumor immunity by restoring T cell effector functions. Together, OVs and ICIs synergize to convert the TME from an immunosuppressive to an immune-active state

The combination of OVs with ICIs offers a promising strategy to amplify antitumor immunity. OV-induced ICD and TME remodeling can enhance T-cell infiltration and activation, while ICIs counteract local immunosuppression, together fostering a more robust and durable clearance of tumor cells [143] (Fig. 3). Numerous clinical trials are currently evaluating combination therapies involving both DNA and RNA oncolytic viruses, as summarized in Tables 3 and 4.

**Table 3:** Clinical trials of DNA oncolytic virus combination therapy

Virus	Biological agent	Combination drugs	Patients	Clinical trial	Clinical trial No
Adv	H101	Lenvatinib, Toripalimab	Advanced Biliary Tract Cancer	II	NCT06919848
	H101	Camrelizumab	Recurrent Cervical Cancer	II	NCT05234905
	H101	Sorafenib	Advanced Hepatocellular Carcinoma	IV	NCT05113290
	H101	FOLFOX	Intrahepatic Mass-forming Cholangiocarcinoma	IV	NCT05124002
	ADV/HSV-tk	Pembrolizumab	Metastatic Triple Negative Breast Cancer and Metastatic Non-Small Cell Lung Cancer	II	NCT03004183
	MEM-288	Nivolumab, Docetaxel	Advanced Solid Tumors	I	NCT05076760
	DNX-2401	Temozolomide	Glioblastoma at First Recurrent	I	NCT01956734
	TILT-123	Avelumab	Solid Tumor (Melanoma and SCCHN) Refractory to or Progressing After Anti-PD(L)1	I	NCT05222932
	TILT-123	Cyclophosphamide and Fludarabine	Melanoma	I	NCT06961786
	TILT-123	Pembrolizumab, or Pembrolizumab and Pegylated Liposomal Doxorubicin	Platinum Resistant or Refractory Ovarian Cancer	I/II	NCT05271318
	Ad/MG1-E6E7	Atezolizumab	HPV-Associated Cancers	I	NCT03618953
	Enadenotucirev	Capecitabine, Radiotherapy	Locally Advanced Rectal Cancer	I	NCT03916510
	MG1-MAGEA3	Pembrolizuma	Previously Treated Metastatic Non-Small Cell Lung Cancer	I/II	NCT02879760
	BioTTT001	SOX and Toripalimab	Peritoneal Metastases From Gastric Cancer	II	NCT06283121
	BioTTT001	Toripalimab and Regorafenib	Liver Metastases From Colorectal Cancer	I	NCT06283134
	VCN-01	Gemcitabine and Abraxane^®^	Advanced Pancreatic Cancer	I	NCT02045589
	VCN-01	Nab-Paclitaxel and Gemcitabine	Metastatic Pancreatic Cancer	II	NCT05673811
	GM103	Pembrolizumab	Locally Advanced, Unresectable, Refractory and/or Metastatic Solid Tumors	I/II	NCT06265025
	ONCOS-102	Balstilimab	Unresectable or Metastatic Cutaneous Melanoma Resistant to Anti-PD-(L)1 Treatment	II	NCT05561491
	OBP-301	Pembrolizumab	Head and Neck Squamous Cell Carcinoma With Inoperable, Recurrent or Progressive Disease	II	NCT04685499
	LOAd703	Gemcitabine and Nab-paclitaxel +/- the Anti-PD-L1 Antibody Atezolizumab	Pancreatic Cancer	I/II	NCT02705196
HSV	T-VEC	Isolated Limb Perfusion with Melphalan and Tumor Necrosis Factor	Advanced Extremity Tumors	I/II	NCT03555032
	T-VEC	Autologous CD1c (BDCA-1)+ Myeloid Dendritic Cells	Melanoma	I	NCT03747744
	T-VEC	Neoadjuvant Chemotherapy and Radiation	Adenocarcinoma of the Rectum	I	NCT03300544
	T-VEC	Preoperative External Beam Radiation Therapy	Locally Advanced Soft Tissue Sarcomas	II	NCT06660810
	T-VEC	Chemotherapy or Endocrine Therapy	Metastatic, Unresectable, or Locoregionally Recurrent HER2-negative Breast Cancer	I	NCT03554044
	T-VEC	Pembrolizumab	Unresectable Stage IIIB to IVM1c Melanoma (MASTERKEY-265)	Ⅲ	NCT02263508
	T-VEC	Hypofractionated Radiotherapy	Cutaneous Melanoma, Merkel Cell Carcinoma, or Other Solid Tumors	II	NCT02819843
	T-VEC	Ipilimumab	Unresected, Stage IIIB-IV Melanoma	I/II	NCT01740297
	T-VEC	Nivolumab	Resectable Early Metastatic (stage IIIB/C/D-IV M1a) Melanoma	II	NCT04330430
	T-VEC	Atezolizumab	Residual Breast Cancer After Standard Neoadjuvant Multii-agent Chemotherapy	I	NCT03802604
	T-VEC	Nivolumab and Trabectedin	Advanced Sarcoma, Including Desmoid Tumor and Chordoma	II	NCT03886311
	T-VEC	Pembrolizumab	Metastatic and/or Locally Advanced Sarcoma	II	NCT03069378
	T-VEC	Dabrafenib and Trametinib	Advanced Melanoma With an Activating BRAF Mutation	I	NCT03088176
	T-VEC	Pembrolizumab	Advanced Melanoma Who Have Progressed on Anti-PD1/L1 Based Therapy	II	NCT02965716
	T-VEC	Nivolumab	Malignant Pleural Effusion	I/II	NCT03597009
	T-VEC	Nivolumab	Refractory T-Cell and NK Cell Lymphomas, Cutaneous Squamous Cell Carcinoma, Merkel Cell Carcinoma, and Other Rare Skin Tumors	II	NCT02978625
	T-VEC	BRAF/MEK inhibitor	Advanced Nodal BRAF Mutant Melanoma	II	NCT03972046
	T-VEC	Paclitaxel	Triple Negative Breast Cancer	I/II	NCT02779855
	T-VEC	Pembrolizumab	Unresectable/Metastatic Stage IIIB-IVM1d Melanoma Who Have Progressed on Prior Anti PD-1 Based Therapy	II	NCT04068181
	T-VEC	Ipilumumab, Nivolumab	Localized Breast Cancer-deleted	I	NCT04185311
	T-VEC	Panitumumab	Locally Advanced Squamous Cell Carcinoma of the Skin	I	NCT04163952
	T-VEC	Pembrolizumab	Advanced Solid Tumors	I/II	NCT02509507
	T-VEC	Pembrolizumab	Recurrent or Metastatic Squamous Cell Carcinoma of the Head and Neck	I	NCT02626000
	T-VEC	Radiotherapy	Locally Advanced Soft Tissue Sarcomas	I/II	NCT04599062
	T-VEC	Atezolizumab	Triple Negative Breast Cancer and Colorectal Cancer With Liver Metastases	I	NCT03256344
	T-VEC	Concurrent Cisplatin & Radiotherapy	Locally Advanced Squamous Cell Carcinoma Of The Head And Neck	III	NCT01161498
	OH2	HX008	Malignant Solid Tumors	I/II	NCT03866525
	OH2	Keytruda	Advanced Solid Tumors	I/II	NCT04386967
	OH2	Capecitabine, Bevacizumab	Advanced Colorectal Cancer	II	NCT05648006
	OH2	HX008	Melanoma	I/II	NCT04616443
	JNJ-87,704,916	Cetrelimab	Advanced Solid Tumors	I	NCT06311578
	RP1	Atezolizumab	Triple-Negative Breast Cancer	I/II	NCT06067061
	RP1	Cemiplimab	Advanced Cutaneous Squamous Cell Carcinoma	II	NCT04050436
	RP1	Nivolumab	Solid Tumors	II	NCT03767348
	RP2	Bevacizumab, Atezolizumab	Locally Advanced Unresectable, Recurrent and/or Metastatic Hepatocellular Carcinoma	II	NCT05733598
	RP2 or RP3	Atezolizumab and Bevacizumab	Advanced Microsatellite Stable and Mismatch Repair Proficient Colorectal Carcinoma	II	NCT05733611
	RP3	Concurrent Chemoradiation Therapy Followed by Nivolumab or combined with Chemotherapy and Nivolumab	Locoregionally Advanced or Recurrent Squamous Cell Carcinoma of the Head and Neck	II	NCT05743270
	HF10	Gemcitabine + Nab-paclitaxel or TS-1	Stage III or IV Unresectable Pancreatic Cancer	I	NCT03252808
	HF10	Ipilimumab	Stage IIIB, IIIC, or IV Unresectable or Metastatic Malignant Melanoma	II	NCT03153085
	T3011	Toripalimab and Regorafenib	Liver Metastases From Colorectal Cancer	I	NCT06283303
	G207	Single 5 Gy Radiation Dose	Children With Recurrent High-Grade Glioma	II	NCT04482933
	ONCR-177	Pembrolizumab	Advanced and/or Refractory Cutaneous, Subcutaneous or Metastatic Nodal Solid Tumors or With Liver Metastases of Solid Tumors	I	NCT04348916
	VG161	Nivolumab	Advanced Pancreatic Cancer	I/II	NCT05162118
	VG161	Nivolumab	Metastatic Gastric Cancer	I/II	NCT06008925
	VG161	Camrelizumab	Advanced Primary Hepatocellular Carcinoma	I/II	NCT06124001
	VG161	Nivolumab	Hepatocellular Carcinoma or Intrahepatic Cholangiocarcinoma	I/II	NCT05223816
VV	ASP9801	Pembrolizumab	Advanced/Metastatic Solid Tumors	I	NCT03954067
	TG6002	5-flucytosine	Recurrent Glioblastoma Patients	I/II	NCT03294486
	Pexa-Vec	Durvalumab, Tremelimumab	Refractory Colorectal Cancer	I/II	NCT03206073
	Pexa-Vec	Sorafenib	Advanced Hepatocellular Carcinoma (HCC) Without Prior Systemic Therapy	III	NCT02562755
	Pexa-Vec	Irinotecan	Metastatic, Refractory Colorectal Carcinoma	I/II	NCT01394939
	TBio-6517	Pembrolizumab	Advanced Solid Tumors	I	NCT04301011
	CF33-hNIS	pembrolizumab or mFOLFOX	Metastatic or Advanced Solid Tumors	I	NCT05346484
	KM1	Chemotherapy	Recurrent or Refractory Ovarian Cancer	I	NCT05684731
	GL-ONC1	Concurrent Cisplatin and Radiotherapy	Locoregionally Advanced Head and Neck Carcinoma	I	NCT01584284
	T601	Oral Flucytosine (5-FC)	Advanced Malignant Solid Tumors	I/II	NCT04226066
	CF33-CD19	Blinatumomab	Advanced or Metastatic Solid Tumors	I	NCT06063317
	BT-001	Pembrolizumab	Cutaneous or, Subcutaneous Lesions or Easily Injectable Lymph Nodes of Metastatic/Advanced Solid Tumors	I/II	NCT04725331
	Olvi-Vec	Platinum-doublet Chemotherapy and Bevacizumab	Women With Platinum-Resistant/Refractory Ovarian Cancer	III	NCT05281471

Adv, adenovirus; HSV, herpes simplex virus; VV, vaccinia virus; SCCHN, squamous cell carcinoma of the head and neck; PD (L) 1, programmed death protein (ligand) 1; HPV, human papilloma virus; BRAF, v-raf murine sarcoma viral oncogene homolog B1

**Table 4:** Clinical trials of RNA oncolytic virus combination therapy

Virus	Biological agent	Combination drugs	Patients	Clinical trial	Clinical trial No
CVA	CAVATAK^®^	Ipilimumab	Uveal Melanoma Metastatic to Liver	I	NCT03408587
NDV	PIN	Anti-PD1	Refractory Advanced Primary Hepatocellular Carcinoma	I	NCT07018518
MV	TMV-018	5-Fluorocytosine (5-FC) or An Anti-PD-1 Checkpoint Inhibitor	Tumors of the Gastrointestinal Tract	I	NCT04195373
VSV	OVV-01	Pembrolizumab or Atezolizumab	Advanced Solid Tumors	I	NCT04787003
	OVV-01	IBR900 Cell Injection	Advanced Malignant Tumors	I	NCT05271279
	Revottack	PD-1 Inhibitor	Advanced Malignant Solid Tumor	I	NCT05644509
RV	Pelareorep	INCMGA00012	Metastatic Triple Negative Breast Cancer	II	NCT04445844
	Pelareorep	Avelumab and Paclitaxel	Breast Cancer Metastatic	II	NCT04215146
	Pelareorep	Dexamethasone, Carfilzomib, and Nivolumab	Relapsed Multiple Myeloma	I	NCT03605719
	REOLYSIN^®^	FOLFIRI and Bevacizumab	KRAS Mutant Metastatic Colorectal Cancer	I	NCT01274624
	REOLYSIN^®^	Chemotherapy and Pembrolizumab	Advanced Pancreatic Adenocarcinoma	I	NCT02620423
	REOLYSIN^®^	Gemcitabine and Cisplatin	Muscle-invasive Transitional Cell Carcinoma of the Bladder	I	NCT02723838
SVV	SVV-001	Nivolumab and Ipilimumab	Poorly Differentiated Neuroendocrine Carcinomas or Well-Differentiated High-Grade (Grade 3) Neuroendocrine Tumors	I	NCT06889493
Alphavirus	M1	SHR-1210 and Apatinib	Advanced/Metastatic Hepatocellular Carcinoma	I	NCT04665362

CVA, coxsackievirus A; NDV, Newcastle disease virus; MV, measles virus; VSV, vesicular stomatitis virus; RV, reovirus; SVV, Seneca Valley virus; PD (L) 1, programmed death protein (ligand) 1

### Combinatorial strategies involving OVs and CTLA-4 inhibitors

CTLA-4 (CD152), a critical immune checkpoint molecule within the immunoglobulin superfamily, negatively regulates T-cell activation and was among the first ICIs approved for clinical use [144, 145]. Genetic ablation of CTLA-4 specifically in Tregs induces systemic lymphoproliferation, fatal T-cell-driven autoimmunity, elevated immunoglobulin E levels, and enhanced antitumor immunity [146]. In conventional T cells, CTLA-4 expression is upregulated upon activation and suppresses immune responses, a mechanism frequently exploited in cancer to promote immune evasion [147]. By binding to CD80 and CD86 on APCs, CTLA-4 attenuates T-cell activation; blockade of this interaction potently reinvigorates antitumor T-cell responses [148, 149], leading to the clinical approval of several CTLA-4-targeting agents.

OVs can remodel the TME and induce robust intratumoral T cell responses, but simultaneously upregulate inhibitory checkpoints such as CTLA-4; thus, combination with CTLA-4 blockade synergistically enhances antitumor immunity by relieving T cell exhaustion and amplifying adaptive immune responses [150]. A promising strategy to synergize oncolytic virotherapy with CTLA-4 blockade involves engineering OVs to express anti-CTLA-4 antibodies within the TME [151]. Ad5/3-D24aCTLA4, an oncolytic Adv encoding a human anti–CTLA-4 monoclonal antibody, has demonstrated enhanced antitumor efficacy in studies comparing replication-competent and replication-deficient variants [152]. Tumoral replication resulted in high local antibody concentrations, effective CTLA-4 pathway blockade, and enhanced T-cell activity, evidenced by increased IL-2 secretion. Activation was selective for T cells from cancer patients, with no effect on those from healthy donors. Critically, antibody levels were significantly higher in tumors than in plasma, where they remained within a safe range, supporting the concept that localized immunomodulation via OVs may minimize systemic toxicity [152].

The armed oncolytic HSV-1 construct RP2, which expresses GM-CSF, a fusogenic protein (GALV-GP R), and an anti–CTLA-4 antibody, demonstrated encouraging preliminary efficacy and safety both as monotherapy and in combination with anti–PD-1 therapy in a phase I trial involving patients with solid tumors (NCT04336241). Sustained partial responses were observed in 50% of patients, including regression in noninjected lesions, underscoring its systemic immunotherapeutic potential [153].

In current clinical practice, combining OVs with established ICIs represents a more common approach, with dosing strategies being optimized to maximize therapeutic benefit [154]. For example, a phase II trial in melanoma (NCT01740297) compared T-VEC combined with ipilimumab versus ipilimumab alone. The combination group exhibited significantly improved outcomes: ORR (35.7% vs. 16.0%; OR = 2.9, *p* = 0.003), durable response rate (33.7% vs. 13.0%), median progression-free survival (13.5 vs. 6.4 months), and 5-year overall survival (54.7% vs. 48.4%) [155]. These results affirm the long-term efficacy and feasibility of combining OVs with CTLA-4 blockade and provide robust clinical support for such strategies in advanced melanoma.

### Combinatorial strategies involving OVs and PD-1/PD-L1 inhibitors

Analogous to CTLA-4 blockade, inhibition of the PD-1/PD-L1 axis enhances antitumor immunity by releasing inhibitory signals that constrain T-cell function. PD-1 (CD279), a type I transmembrane glycoprotein belonging to the CD28 receptor superfamily, contains two cytoplasmic tyrosine-based motifs that undergo phosphorylation upon ligand engagement [156, 157]. This immune checkpoint is expressed on T cells, B cells, and NK cells, and plays a critical role in maintaining immune homeostasis [158–160]. Its ligands, PD-L1 and PD-L2, are frequently overexpressed on both immune and tumor cells, including melanoma, lung, and breast cancers, enabling tumors to evade immune destruction [161–163]. Binding of PD-1 to its ligands suppresses T-cell activation and promotes an immunosuppressive TME [164]. Tumor cells exploit this pathway to evade immune surveillance and clearance [136]. Blockade of this interaction reverses T-cell exhaustion and restores antitumor reactivity, forming a cornerstone of modern cancer immunotherapy [165]. Numerous PD-1/PD-L1 inhibitors, such as cemiplimab (Libtayo) [166], nivolumab (Opdivo) [167], avelumab (Bavencio) [168], durvalumab (Imfinzi) [169], and pembrolizumab (Keytruda) [170], are now widely used clinically with marked success across cancer types [171].

OVs can further potentiate PD-1/PD-L1 blockade by reversing local immunosuppression and promoting T-cell infiltration. For instance, although the oncolytic HSV-OVH exhibits antitumor activity, it often fails to achieve complete tumor clearance and can induce PD-1 upregulation, limiting T-cell cytotoxicity [82]. To address this, YST-OVH was engineered to express a single-chain variable fragment (scFv) against human PD-1. This virus demonstrated robust replication and scFv production in vitro and in vivo, leading to enhanced CD8⁺ T cell infiltration, reduced T-cell exhaustion, and establishment of memory responses. Combination with anti–CTLA-4 or anti–TIM-3 antibodies further augmented efficacy [150]. Similarly, the combination of oncolytic VV-JX-594 with a PD-1 inhibitor reduced liver toxicity compared to ICI monotherapy [172]. Innovative delivery approaches also show promise: Zhu et al. developed PD-1/Al@OV, an OV coated with an anti–PD-1 antibody and alendronate, which enhanced T-cell activity and reduced phagocytosis by TAMs, improving glioblastoma treatment outcomes [173].

Clinical evidence continues to support the synergy between OVs and PD-1/PD-L1 inhibitors [174]. In a phase II study involving patients with anti–PD-1–resistant advanced melanoma (NCT04068181), T-VEC combined with pembrolizumab yielded an ORR of 40–46.7% in patients who experienced recurrence after adjuvant therapy, although the efficacy was limited in those with primary resistance [175]. Another study addressed post-incomplete radiofrequency ablation (iRFA) immunosuppression using oHSV2-mGM combined with anti–PD-1 therapy, which significantly improved complete response rates in mouse models by countering residual tumor growth [176]. In a phase I trial for mucosal melanoma with liver metastases (NCT04206358, NCT05070221), intrahepatic oHSV2-hGMCSF combined with axitinib and the anti–PD-1 antibody pucotenlimab resulted in an ORR of 26.7% and disease control in 46.7% of patients, with biopsies showing complete tumor clearance and robust tumor-infiltrating lymphocyte (TIL) infiltration in some cases [177]. Notably, virus-based therapeutic strategies have also demonstrated promising efficacy in non-solid tumor settings such as malignant ascites. The chimeric orthopoxvirus CF33-hNIS, when administered intraperitoneally in combination with anti-PD-L1 blockade, exhibits robust antitumor activity in gastric cancer–derived peritoneal metastasis, significantly enhancing CD3⁺ and CD8⁺ T cell infiltration within the peritoneal cavity and TME, promoting the generation of effector and central memory T cells, and inducing durable antitumor immunity capable of rejecting tumor rechallenge, thereby effectively reprogramming the immunosuppressive ascites microenvironment [178]. Future studies should further optimize these strategies through rational combinations with immune checkpoint blockade and myeloid-targeting therapies to enhance therapeutic durability and overcome residual immunosuppression within the ascites microenvironment.

The multi-cytokine–armed oncolytic herpesvirus VG161, which expresses IL-12, IL-15, IL-15Rα, and a PD-1–PD-L1 blocking fusion protein, demonstrated immune activation in a phase I trial (NCT04806464) [179]. Transient decreases in peripheral T and NK cells at 24 h were followed by recovery and expansion by days 15–28, suggesting immune cell trafficking to tumors. Increased CD8⁺ and CD4⁺ T cell infiltration and enhanced NK–T cell interactions in non-injected lesions confirmed systemic immune activation. Cytokine surges (IL-6, TNF, and IFN) at 24 h indicated robust innate immune activation and ICD [180].

### Combinatorial strategies involving OVs and other ICIs

A new generation of ICI-targeting molecules beyond CTLA-4 and PD-1/PD-L1 is under active development, showing significant potential as powerful complements or alternatives to existing immunotherapies [181–183]. The combination of OVs with these mechanistically diverse ICIs represents an emerging and promising strategy for cancer treatment [184, 185].

Among these novel targets, TIM-3, an immune checkpoint receptor expressed on immune cells, mediates immunosuppressive interactions through ligands such as galectin-9 [139]. In parallel, strategies targeting the TIGIT pathway have also gained attention. Unlike OVs engineered to directly express TIGIT-blocking molecules, Zhang et al. developed a recombinant oncolytic Adv, Ad5sPVR, which secretes soluble poliovirus receptor (sPVR). This soluble receptor binds with high affinity to TIGIT, effectively blocking the immunosuppressive PVR/TIGIT axis while simultaneously activating the CD226-mediated costimulatory pathway. This dual action significantly enhances the infiltration and function of CD8⁺ T cells and NK cells and promotes IFN-γ secretion [186]. Building on this concept, the same group constructed Ad5sPD1PVR, an Adv expressing a fusion protein (sPD1PVR) that concurrently blocks the PD-1/PD-L1 inhibitory pathway and activates CD226 costimulation. This engineered virus synergistically induces CD8⁺ T cell-dependent, long-lasting antitumor immunity [187].

Additionally, the combination of TIM-3 blockade with myxoma virus (MYXV)-based oncolytic virotherapy has demonstrated remarkable efficacy [188]. The robust antitumor immune response triggered by MYXV is further amplified by TIM-3 inhibition, which reprograms the TME toward a proinflammatory state. This combination resulted in complete tumor eradication in models where monotherapies proved ineffective [189].

A cutting-edge approach involves arming OVs with bispecific antibodies that engage both immune checkpoint targets and T cells, representing a prominent frontier in cancer immunotherapy [190]. For example, HSV-1DKO-B7H3nb/CD3 is an engineered oHSV encoding a bispecific antibody targeting B7H3 and CD3. B7H3 is frequently overexpressed in the TME and facilitates tumor immune evasion; its blockade helps restore antitumor immunity [191]. Meanwhile, CD3 engagement amplifies TCR signaling, enhancing T-cell activation [192]. Compared to control virus, HSV-1DKO-B7H3nb/CD3 provoked stronger antitumor immune responses with increased T-cell infiltration. Furthermore, it elevated numbers of NK cells and effector CD8⁺ T cells while reducing immunosuppressive populations such as Tregs, MDSCs, and M2 macrophages [193]. Similarly, oHSV2 engineered to express PD-L1/CD3 bispecific antibodies can not only induce direct oncolysis but also activate PBMC-derived T cells by bridging CD3 with PD-L1 on tumor cells, thereby triggering MHC-independent cytotoxicity and enhancing antitumor efficacy in vitro and in vivo [194].

The combination of OVs and ICIs synergistically enhances antitumor immunity by leveraging the dual mechanisms of OV-mediated immunogenic cell death and TME remodeling, together with ICI blockade of inhibitory pathways such as CTLA-4 and PD-1/PD-L1. This approach promotes robust T-cell infiltration and activation, counteracts local immunosuppression, and achieves durable systemic responses. Clinical trials combining OVs like T-VEC with ipilimumab or pembrolizumab demonstrate significantly improved outcomes in melanoma. Importantly, therapeutic efficacy is influenced not only by the choice of checkpoint targets but also by the mode of ICI delivery. OV-mediated intratumoral expression of ICIs enables spatially restricted and sustained immune modulation within the TME, thereby enhancing local efficacy while potentially minimizing systemic toxicity. In contrast, systemically administered ICIs provide broad immune activation across both primary and metastatic sites but are more frequently associated with immune-related adverse events and may exhibit limited efficacy in poorly inflamed (“cold”) tumors. Accordingly, engineering OVs to express ICIs locally represents a rational strategy to optimize the therapeutic window of checkpoint blockade. Beyond conventional ICIs, next-generation approaches incorporating targets such as TIM-3 and TIGIT, as well as bispecific antibodies, are being explored to further amplify immune activation. In parallel, emerging studies are investigating the integration of OV-based therapies with cellular immunotherapies, including CAR-T cells and TCR-engineered T cells, to enhance tumor specificity and overcome barriers associated with solid tumors.

## OVs in combinations with cell therapy: mechanisms and clinical applications

Following the development of strategies that employ antibodies to block immunosuppressive signals in conjunction with OVs, a major advancement in cancer immunotherapy has been the direct enhancement of immune cells’ inherent capacity to recognize and eliminate tumors. As central effectors of the immune response, immune cells play a critical role in regulating and executing antitumor immunity, rendering them invaluable agents in cancer treatment [195, 196]. The successful induction and maintenance of potent antitumor immune responses within the complex TME rely on coordinated interactions among diverse immune cell types, including T cells, NK cells, DCs, and macrophages [197–200]. However, the highly immunosuppressive nature of the TME often results in dysregulated immune cell distribution, numerical deficits, and impaired signaling, thereby hampering effective antitumor immunity [201–203].

To overcome these limitations, immune cell-based therapies have emerged as a forefront therapeutic paradigm. These approaches typically involve the isolation of a patient’s immune cells, their ex vivo modification and expansion, and subsequent reinfusion, with the goal of enhancing tumor-specific targeting and amplifying endogenous immune responses [204]. Among these, T cell–centric therapies occupy a central role due to their capacity for specific antigen recognition, potent cytotoxicity, immunological memory, and favorable safety profile [205]. Current leading T cell–based modalities include chimeric antigen receptor T cell (CAR-T) therapy [206], T cell receptor–engineered T cell (TCR-T) therapy [207], and TIL therapy [208]. Beyond T cell therapies, other immune effector cells have also been harnessed, such as cytokine-induced killer (CIK) cells [209], DC-based vaccines [210], and more recently, CAR-NK cells [211] and CAR-macrophages (CAR-M) [212]. Each of these strategies employs distinct mechanisms to achieve precise tumor recognition and elimination.

**Fig. 4:**
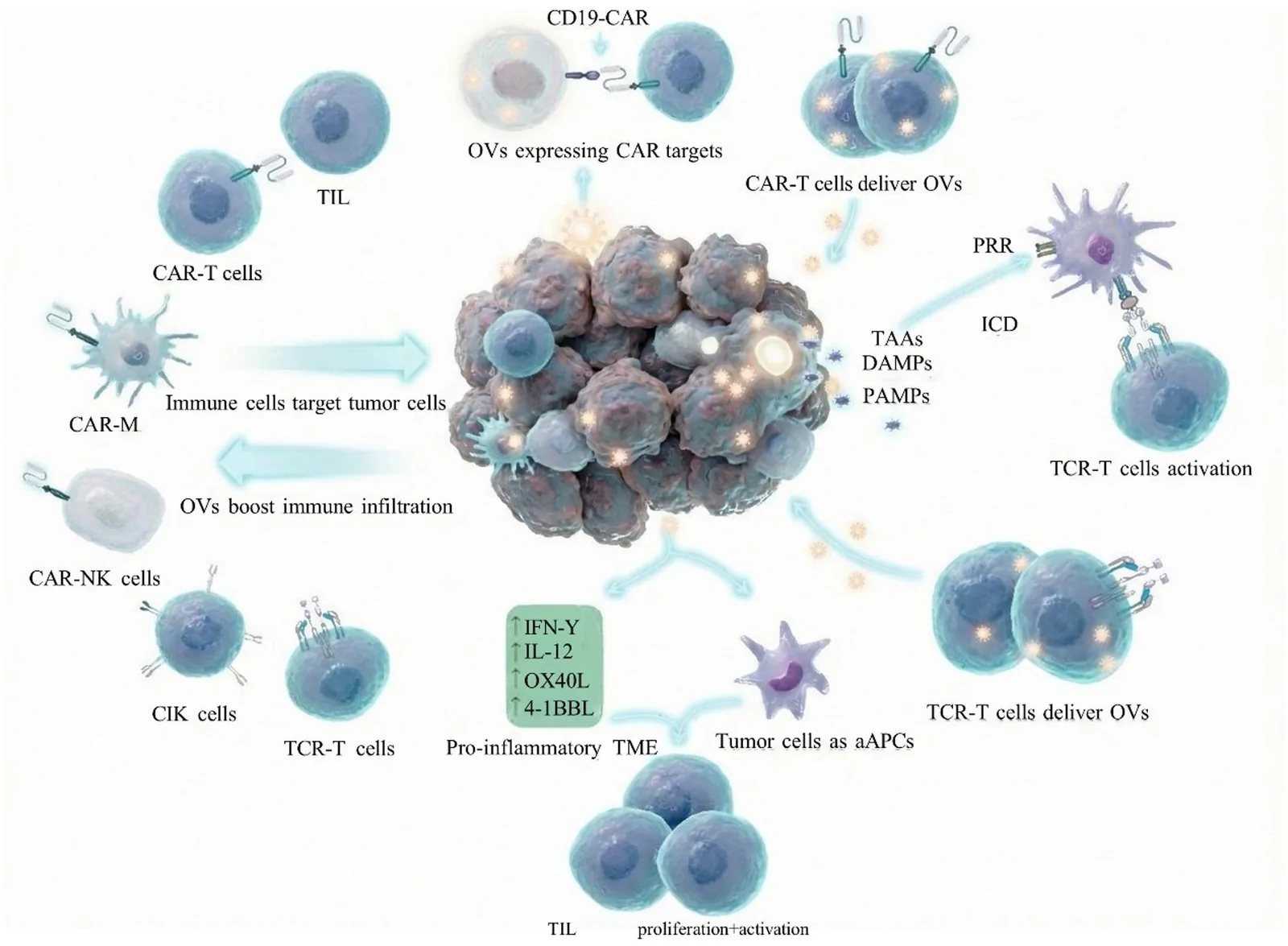
Synergistic mechanisms between OVs and cell therapy. Schematic illustration showing how OVs cooperate with multiple forms of adoptive cell therapy, including CAR-T, TCR-T, CAR-NK, CAR-M, CIK cells, and TILs, to enhance antitumor immunity. OVs infect and lyse tumor cells, releasing TAAs, DAMPs, and PAMPs, which engage PRRs on antigen-presenting cells and promote ICD. Viral infection reshapes the TME into a pro-inflammatory state characterized by increased IFN-γ, IL-12, OX40L, and 4-1BBL expression, thereby supporting TIL expansion and T cell priming. Engineered immune cells, including CAR-T and TCR-T cells, can also serve as targeted delivery vehicles for OVs, enhancing intratumoral viral distribution. Conversely, OVs augment immune-cell infiltration and sensitize tumor cells to immune-mediated killing. Collectively, these reciprocal interactions strengthen lymphocyte activation, effector function, and tumor recognition, resulting in amplified and durable antitumor responses. OVs: oncolytic viruses, CAR-T cells: chimeric antigen receptor T cells, TCR-T cells: T-cell receptor–engineered T cells, CAR-NK cells: chimeric antigen receptor natural killer cells, CAR-M cells: chimeric antigen receptor macrophages, CIK cells: cytokine-induced killer cells, TILs: tumor-infiltrating lymphocytes, TAAs: tumor-associated antigens, DAMPs: danger-associated molecular patterns, PAMPs: pathogen-associated molecular patterns, PRR: pattern recognition receptor, ICD: immunogenic cell death, TME: tumor microenvironment, IFN-γ: interferon-γ, IL-12: interleukin-12, OX40L: OX40 ligand, 4-1BBL: 4-1BB ligand

Despite their considerable promise, these advanced cellular immunotherapies face challenges including high manufacturing complexity, cost, and risks such as T cell exhaustion, off-target toxicity, and iatrogenic immunosuppression [213]. In response, researchers are increasingly investigating combination regimens that incorporate OVs with cell therapies (Fig. 4). Such integrative strategies seek to modulate the TME, enhance immune cell infiltration and functionality, and ultimately improve treatment outcomes, offering new avenues for safer and more effective cancer therapies.

### Enhancing CAR-T cell efficacy in solid tumors via OVs

CAR-T-cell therapy represents a paradigm-shifting therapy in the treatment of hematological malignancies. This approach utilizes genetic engineering to modify a patient’s own T cells, enabling them to express synthetic CARs that confer novel tumor-targeting capabilities [214–216]. These synthetic receptors exhibit high specificity for antigens present on the surface of cancer cells [217]. Upon antigen engagement, CAR signaling activates T cells, unleashing potent cytotoxic responses and enabling precise and efficient destruction of malignant cells [218–220]. To date, regulatory agencies in several countries have approved multiple CAR-T-cell therapeutics, including Breyanzi, Abecma, Yescarta, Tecartus, and Kymriah, marking substantial progress in the field [221]. However, the efficacy of CAR-T cells in solid tumors remains limited, primarily due to poor tumor infiltration, impaired persistence and proliferation within the immunosuppressive TME, and a lack of truly tumor-specific target antigens [222].

Combining CAR-T cells with OVs offers a promising strategy to overcome these barriers and enhance therapeutic outcomes [223–226]. Beyond promoting tumor infiltration and remodeling the immunosuppressive TME, OVs can also function as immunological adjuvants that actively reprogram CAR-T cell responses. Upon infection, OVs introduce viral antigens and inflammatory signals that engage endogenous TCR signaling in CAR-T cells, thereby generating dual-specific (CAR/TCR) T cells with enhanced expansion, effector function, and long-term persistence. This antigen-driven reactivation not only amplifies cytokine production and tumor cell killing but also supports sustained in vivo maintenance and recall responses following viral re-exposure, collectively improving CAR-T-cell durability and antitumor efficacy [227]. Mechanistically, these effects can be further leveraged through strategies that enhance T-cell recruitment and positioning within tumors. Transcriptomic analysis of T cells isolated from glioblastoma patients revealed high expression of C-X-C chemokine receptor (CXCR) 3. Correspondingly, an oncolytic Adv engineered to express the CXCR3 ligand chemokine (C-X-C motif) ligand (CXCL) 11 (oAd-CXCL11) was shown to markedly enhance CAR-T-cell migration and infiltration into glioblastoma models. oAd-CXCL11 monotherapy not only exerted significant antitumor effects but also favorably remodeled the TME, increasing the infiltration of CD8⁺ T cells, NK cells, and M1 macrophages while reducing the numbers of MDSCs, Tregs, and M2 macrophages. The combination of oAd-CXCL11 with CAR-T cells further increased antitumor efficacy and T-cell infiltration [228].

Another innovative strategy involves using OVs to deliver artificial CAR targets directly to tumors. Park et al. demonstrated that OV-infected tumor cells could be engineered to express CD19, enabling CD19-directed CAR-T cells to recognize and lyse these tumor cells effectively [229]. Similarly, VV infection induces the expression of the viral A56 protein on the membrane of tumor cells [230]. Capitalizing on this, Cho et al. developed A56-specific CAR-T cells (A56CAR-T), which exhibited potent killing activity against VV-infected tumor cell lines and, in combination with oncolytic VV and hydroxyurea, significantly reduced tumor volume and extended survival in murine models [231].

CAR-T cells themselves can also serve as delivery vehicles for OVs. Studies show that CAR-T cells can be infected with oncolytic HSV-1 without loss of function. In orthotopic glioblastoma models, HSV-1-loaded CAR-T cells successfully delivered the virus to tumor sites, enhancing T-cell infiltration and prolonging survival. In bilateral subcutaneous tumor models, intravenous infusion of these carrier CAR-T cells led to significant inhibition of tumor growth at both sites, whereas intratumoral virus injection only affected locally injected tumors [232].

However, certain OVs may inadvertently impair CAR-T cell function. For instance, VSVmIFNβ, an oncolytic VSV expressing IFN-β, can induce chemokine expression and recruit CAR-T cells into tumors, yet the combination failed to improve tumor control. This was attributed to IFN I–driven apoptosis and upregulation of inhibitory receptors on T cells, resulting in depletion of both CAR-T cells and conventional T cells. Notably, CAR-T cells engineered with defective IFN receptors resisted these deleterious effects and exhibited enhanced antitumor activity in combination with VSVmIFNβ [233]. Although OV-mediated immune activation could theoretically increase the risk of cytokine release syndrome, the localized replication of OVs within tumors may help confine inflammatory responses to the TME and potentially reduce systemic cytokine exposure compared with conventional CAR-T-cell therapy. Nevertheless, this possibility remains to be formally validated. These findings underscore the need for further investigation into the molecular and immunological interactions between OVs and CAR-T cells to optimize combination strategies and advance their clinical translation.

### TCR-engineered T cells and OVs: expanding targetable antigens

In contrast to CAR-T cell technology, which is restricted to surface antigen recognition, TCR-T therapy involves genetically modifying T cells to express exogenous, antigen-specific TCRs, thereby conferring the ability to recognize specific peptide antigens [234–238]. A key advantage of TCR-T cells lies in their capacity to target epitopes derived from both membrane and intracellular proteins, which are presented by major histocompatibility complex (MHC) molecules [239]. Upon encounter with cancer cells displaying cognate antigen–MHC complexes, the introduced TCR activates downstream signaling pathways that trigger T cell proliferation, differentiation, and cytotoxic effector functions [240]. TCR-T technology has evolved through several generations of refinement [241], and achieved a notable clinical milestone with the FDA approval of afamitresgene autoleucel (formerly ADP-A2M4) for advanced synovial sarcoma, representing a significant advance for TCR-T therapy in solid tumors [242].

Despite these advances, the efficacy of TCR-T therapy is frequently compromised in solid tumors by tumor-intrinsic immune evasion mechanisms, particularly the downregulation of MHC-I expression and defects in antigen processing and presentation machinery, which impair TCR recognition of tumor cells [243]. OVs can potentiate TCR-T therapy through multiple mechanisms. Following infection of tumor cells, OVs drive intracellular replication and express viral antigens, stimulating de novo T cell responses against both viruses and TAAs [244]. These activated T cells, via their endogenous or engineered TCRs, recognize and eliminate OV-infected or TAA-expressing tumor cells, amplifying antitumor immunity. Importantly, OV infection can counteract tumor immune evasion by restoring antigen presentation capacity. Viral sensing pathways induce type I interferons and proinflammatory cytokines, which upregulate MHC-I expression, β2-microglobulin, and antigen-processing machinery such as TAP, thereby converting MHC-low tumor cells into recognizable targets for TCR-T cells. In addition, OV-mediated oncolysis promotes the release of tumor-associated antigens and DAMPs, facilitating DC-mediated cross-presentation and further enhancing TCR-dependent immune recognition.

Beyond merely priming antitumor responses, OVs have shown compelling synergy in combination with TCR-T cells. For instance, in models of Ewing sarcoma (EwS), the oncolytic Adv XVir-N-31 synergized powerfully with TCR-transgenic CD8⁺ T cells, yielding robust antitumor activity in vitro and in vivo [245]. The combination not only directly lysed tumor cells but also induced ICD, promoting the release of DAMPs and cytokines that support DC maturation and antigen presentation. XVir-N-31 infection also remodeled the TME, reducing immunosuppressive factors and enhancing phagocytic activity in macrophages and DCs. Together, these effects augmented the expansion, survival, and effector function of TCR-T cells [245]. Collectively, these coordinated effects reinforce antigen presentation pathways, sustain MHC-dependent tumor recognition, and thereby directly address a central limitation of TCR-T therapy in solid tumors.

TCR-T cells can also serve as carriers to improve OV delivery and safety. VSV, an attractive yet challenging OV candidate, often exhibits limited systemic dissemination and dose-limiting toxicities [246]. TCR-T cells can be productively infected with VSV while shielding the virus from neutralizing antibodies. In murine models, TCR-T cells loaded with VSV mediated faster and more potent tumor cell killing compared to free virus or T cells alone. Moreover, cell-associated VSV delivery reduced systemic toxicity, highlighting a promising strategy to enhance the therapeutic index of oncolytic virotherapy [247].

### Remodeling the TME with OVs to boost TIL therapy

TILs are a heterogeneous population of T cells that naturally colonize tumor tissues and possess the ability to recognize patient-specific neoantigens and TAAs [248]. TIL therapy involves surgical resection of tumor tissue, isolation and large-scale ex vivo expansion of these lymphocytes, and subsequent reinfusion into the patient to mount a potent antitumor immune response [249, 250]. TILs recognize antigens presented by MHC class I molecules via their TCRs, with activation signals transduced through the CD3 complex [251]. Upon activation, TILs secrete cytokines such as IFN-γ and TNF-α and release cytotoxic molecules, including perforin and granzymes, directly inducing apoptosis in tumor cells [252–254]. The clinical potential of TIL therapy has been underscored by the recent FDA approval of lifileucel (Amtagvi) for patients with advanced or unresectable melanoma following progression on ICIs and targeted therapies [255]. However, broader application is limited by the immunosuppressive TME, logistical challenges in manufacturing, and high associated costs [256]. OVs offer a promising strategy to overcome these limitations by remodeling the TME and enhancing TIL function [257, 258]. For example, OV-OX40L/IL12, an engineered oncolytic HSV expressing an OX40 ligand and IL-12, can reprogram tumor cells into artificial APCs (aAPCs). This transformation promotes TIL proliferation and activation, as evidenced by increased IFN-γ secretion and enhanced tumoricidal activity. In both patient-derived xenograft and immunocompetent murine models, the combination of OV-OX40L/IL12 with TILs induced complete or near-complete tumor regression, augmented intratumoral immune activation, and skewed macrophage polarization toward the antitumor M1 phenotype [259].

Similarly, in the context of hepatocellular carcinoma, the combination of an OV expressing 4-1BBL and IL-15 (OV-4-1BBL/IL15) with TIL therapy enhanced antitumor immunity. This regimen upregulated antigen presentation machinery on infected tumor cells, boosted TIL-mediated killing, reduced tumor volume, and established durable immune memory. Additionally, it conferred APC-like properties on tumor cells, promoted T cell activation, and redirected TAMs toward an immunostimulatory phenotype [260].

A systematic comparison of four OV platforms, Adv, VV, HSV, and RV, for enhancing TIL therapy revealed that the adenoviral vector TILT-123 (also known as ONCOS-102) performed most effectively. TILT-123 not only significantly reduced tumor volume and improved survival but also, when combined with TILs, achieved a complete response rate of 62.5%, substantially outperforming other OVs and control therapies. This combination also fostered robust antitumor immune memory. Notably, TILs themselves can serve as cellular vehicles for delivering TILT-123 to tumor sites, further enhancing local virus propagation and antitumor efficacy [261]. An ongoing clinical trial (NCT04217473) is currently evaluating the safety and efficacy of TILT-123 in combination with TIL therapy in human patients [262].

### OV Synergy with innate immune cells: NK cells, DCs, and macrophages

Beyond their well-documented synergies with T cells, OVs are increasingly being explored in combination with innate immune cells, including NK cells, DCs, macrophages, and CIK cells, offering innovative strategies to enhance antitumor immunity and achieve substantial therapeutic benefits.

Peripheral blood mononuclear cells (PBMCs) cultured ex vivo with cytokines such as IL-2 can differentiate into CIK cells, a heterogeneous population exhibiting potent non-MHC-restricted cytolytic activity against tumor cells [263, 264]. Owing to their robust antitumor properties, CIK cells have been widely adopted in clinical practice as a form of adoptive immunotherapy [265]. To improve localized delivery and reduce off-target effects, Du et al. co-administered CIK cells with an oncolytic Adv engineered to express IL-12 and IL-15 (CRAd-IL12-IL15) using an injectable hydrogel system. This formulation minimized the dispersal of both virus and cells to non-tumor tissues such as the liver, enabled sustained release, dampened anti-adenoviral immune responses, and promoted potent and persistent antitumor immunity within the tumor following a single injection [266].

DCs play a pivotal role in antitumor immunity through their superior capacity for antigen uptake, processing, and presentation, and their ability to prime and regulate T-cell responses [267]. These attributes make them attractive agents for cancer immunotherapy, including DC-based vaccines [268]. OVs enhance this approach by selectively lysing tumor cells and facilitating the release and presentation of tumor antigens. In one study, the combination of the OV M1 (OVM) with a DC vaccine significantly delayed tumor progression and improved survival [269]. Mechanistically, OVM was shown to downregulate the expression of signal regulatory protein α (SIRPα) on DCs and CD47 on tumor cells, thereby disrupting the CD47–SIRPα “don’t eat me” axis and relieving the inhibition of DC phagocytosis. Furthermore, OVM increased PD-L1 expression on DCs, and the addition of PD-L1 blockade synergistically enhanced the efficacy of combination therapy [269].

NK cells mediate direct tumor cell killing through the release of perforin and granzymes, which permeabilize target cell membranes and induce apoptosis [270–273]. Genetic engineering approaches have been employed to augment the antitumor activity of NK cells, leading to novel modalities such as chimeric antigen receptor NK (CAR-NK) cell therapy [274–276]. The combination of an IL-15/IL-15Rα–expressing oncolytic HSV-1 (OV-IL15C) with EGFR-targeting CAR-NK cells has demonstrated encouraging results. In glioblastoma models, OV-IL15C potently suppressed tumor growth and extended survival. When used in conjunction with CAR-NK cells, it promoted enhanced infiltration and activation of both NK cells and CD8⁺ T cells in the brain TME and improved the persistence of CAR-NK cells [277]. The combination of PD-L1-targeted CAR macrophages and a CD47 antibody-armed oncolytic adenovirus synergistically enhanced macrophage phagocytosis and CD8⁺ T cell-mediated antitumor immunity, resulting in improved therapeutic efficacy in solid tumors [185, 278, 279].

The combination of OVs with cell therapies represents a promising strategy to overcome the limitations of adoptive cell transfer in solid tumors (Table 5). OVs enhance CAR-T cell trafficking and cytotoxicity through localized chemokine expression and antigen delivery, facilitate TCR-T cell expansion and function by promoting antigen presentation and altering the immunosuppressive TME, and significantly boost TIL activity via engineered immunostimulatory transgenes. Furthermore, OVs synergize with innate immune cells, including NK cells, DCs, and CIK cells, by improving their activation, persistence, and tumor-targeting capabilities. These combinations collectively promote a pro-inflammatory TME, enhance immune cell infiltration and function, and establish durable antitumor immunity. Building on these advances in combining OVs with cellular agents, the next section explores their synergies with cancer vaccines, which aim to further amplify antigen-specific immune priming and systemic antitumor responses.

**Table 5:** Comparison of OVs combined with different cell therapies

Cell therapy	Core mechanism	Major limitations	How OVs overcome limitations
CAR-T cells	Engineered T cells recognize tumor antigens and exert cytotoxicity	Poor infiltration in solid tumors	Enhance tumor inflammation
		Antigen heterogeneity	Promote T cell trafficking
		T cell exhaustion	Reduce immunosuppression
			Provide synthetic antigens
TCR-T cells	TCR recognizes peptide-MHC complexes	MHC restriction (HLA-dependent)	Enhance antigen presentation (↑MHC, TAA release)
		Low antigen presentation in tumors	Remodel TME to reduce immunosuppression
		Immunosuppressive TME	Enhance TCR-T expansion and effector function
		Limited persistence and expansion in solid tumors	Enable cell-mediated OV delivery to improve tumor targeting and reduce systemic toxicity
TILs	Expansion of endogenous tumor-reactive T cells	Immunosuppressive TME	Remodel TME
		Functional exhaustion and limited activation of TILs	Enhance TIL activation and proliferation
		Logistical challenges in manufacturing (tumor resection, ex vivo expansion)	Increase antigen presentation and confer APC-like properties to tumor cells
		High cost	Boost TIL cytotoxicity and cytokine production
			Promote immune memory formation
			Enable TILs as carriers to enhance OV delivery and intratumoral spread
NK cells	Innate cytotoxicity independent of antigen specificity	Limited persistence and expansion	Enhance NK cell infiltration via induction of chemokines
		Insufficient infiltration into solid tumors	Improve activation and cytotoxicity
		Immunosuppressive TME inhibiting NK activation	Increase persistence of NK/CAR-NK cells
			Remodel TME to support NK function
			Promote cross-talk with adaptive immunity
DC-based therapy	Antigen presentation and T cell priming	Limited antigen availability	Induce tumor lysis to release TAAs
		Impaired maturation and function in TME	Enhance DC maturation and antigen presentation
		Inhibitory signals restricting phagocytosis	Disrupt inhibitory pathways to promote phagocytosis
			Increase immune activation
M-based therapy	M reprogramming (M2→M1) to restore antitumor function	Predominant M2-like polarization in TME	Reprogram macrophages toward M1 phenotype
		Suppression of antitumor immunity	Enhance phagocytic activity
		Support of tumor growth and immune evasion	Reduce immunosuppressive signaling in TME
			Promote production of pro-inflammatory cytokines
			Support activation of other immune cells

OVs, oncolytic viruses; CAR, chimeric antigen receptor; TCR, T-cell receptor-engineered; MHC, major histocompatibility complex; TAA, tumor-associated antigen; TME, tumor microenvironment; TILs, tumor-infiltrating lymphocytes; APC, antigen-presenting cell; NK, natural killer; DC, dendritic cell; M, macrophage

## Synergistic combinations of OVs and cancer vaccines for antitumor immunity

**Fig. 5:**
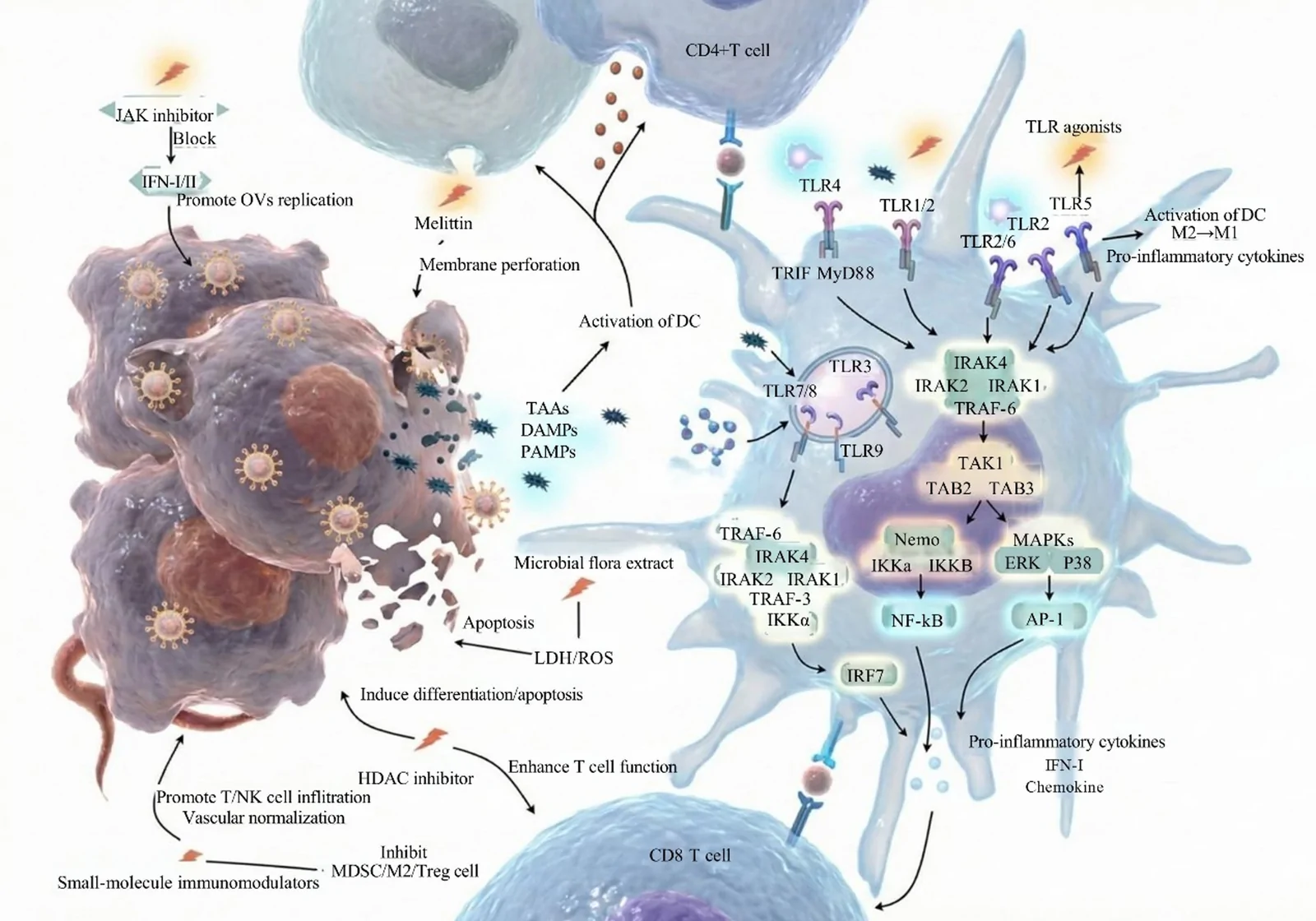
OVs combined with tumor vaccines/small-molecule immunomodulators for tumor therapy. OVs selectively infect and lyse tumor cells, leading to the release of TAAs, DAMPs, and PAMPs, which activate DCs and promote antitumor T-cell responses. Tumor vaccines further enhance DC activation and antigen presentation through TLR signaling pathways, leading to downstream activation of NF-κB, IRF7, and AP-1 transcription factors that induce pro-inflammatory cytokines, IFN-I, and chemokines. Small-molecule immunomodulators augment OV therapy through multiple mechanisms: JAK inhibitors enhance viral replication by blocking IFN-I/Ⅱ responses; HDAC inhibitors promote tumor differentiation and apoptosis while boosting T/NK cell infiltration; TLR agonists and microbial flora extracts stimulate DC activation and reprogram macrophages from the M2 phenotype to the M1 phenotype; and other agents support vascular normalization and reduce immunosuppressive MDSC/M2/Treg populations. Together, these multimodal strategies synergistically enhance CD8⁺ and CD4⁺ T cell activation, resulting in a more inflamed and immunoreactive TME. JAK: Janus kinase, HDAC: histone deacetylase, LDH: lactate dehydrogenase, ROS: reactive oxygen species, TLR: toll-like receptor, TRIF: TIR domain-containing adaptor-inducing interferon-β, MyD88: myeloid differentiation primary response gene 88, TRAF: TNF receptor-associated factor, IRAK: interleukin-1 receptor-associated kinase, IKK: inhibitor of κB kinase, TAK: TGF-β-activated kinase 1, IRF: interferon regulatory factor, TAB: TAK1 binding protein, Nemo: NF-κB essential modulator, MAPKs: mitogen-activated protein kinases, ERK: extracellular signal-regulated kinase, AP-1: activator protein 1, NF-κB: nuclear factor kappa B

Cancer vaccines expose the host immune system to tumor-specific antigens, thereby enabling their recognition, thereby enabling precise targeting and attack of cancer cells [280–284]. This mechanism gives them immense potential in cancer prevention and treatment. For example, by effectively blocking infection by multiple known carcinogenic viral strains, the human papillomavirus (HPV) vaccine successfully prevents various types of HPV-induced malignancies, including cervical cancer. Consequently, it has been incorporated into public health systems in many countries, especially those that target female populations [285–287]. For diagnosed cancer patients, tumor vaccines can activate antitumor responses and maintain long-term memory to eliminate residual tumors and prevent cancer recurrence [288]. On the basis of their biochemical characteristics, cancer vaccines can be categorized into various types, including tumor cell vaccines [289], protein and peptide vaccines [290, 291], nucleic acid vaccines [292], viral vector vaccines [293], and DC vaccines [294]. OVs themselves can function as tumor vaccines. Using OVs as vectors to carry tumor-specific antigens or immunostimulatory molecules to prepare tumor vaccines enables efficient local delivery of these antigens within tumors and immune system activation [295, 296] (Fig. 5). For example, Galanis et al. [297] reported phase I clinical results for an oncolytic MV expressing carcinoembryonic antigen (MV-CEA) in patients with recurrent glioblastoma (NCT00390299). This study demonstrated the safety and efficacy of MV-CEA in a patient cohort, with a median OS of 11.6 months and a one-year survival rate of 45.5%.

### Protein and peptide-based vaccines in conjunction with OVs

The combination of OVs with cancer vaccines represents a promising therapeutic strategy that significantly enhances treatment efficacy. This synergistic approach not only amplifies the immunogenicity of tumor vaccines but also capitalizes on the oncolytic activity of OVs to remodel the TME, fostering conditions conducive to immune-mediated tumor clearance [298]. These modifications facilitate improved immune cell infiltration and immune-mediated tumor cell elimination, underscoring the considerable potential and clinical applicability of such combination regimens [299].

Protein-based vaccines are regarded as particularly advantageous owing to their favorable safety profiles and capacity to target multiple TAAs, allowing for broad and flexible therapeutic applications [300, 301]. For instance, Das et al. demonstrated that a combination regimen incorporating the self-adjuvanting protein vaccine KISIMA with a recombinant oncolytic VSV expressing TAAs (VSV-GP-TAA) robustly induced antitumor immunity [302]. This combination altered the immune landscape of the TME, substantially increasing the frequency and absolute numbers of antigen-specific CD8⁺ T cells in peripheral blood. These T cells exhibited persistence and differentiated into long-lived memory populations, contributing not only to enhanced therapeutic outcomes but also to prolonged survival [302].

The Toll-like receptor (TLR) signaling pathway plays a pivotal role in vaccine immunogenicity by recognizing PAMPs/DAMPs, driving DC maturation and facilitating antigen cross-presentation. These processes are critical for the induction of potent tumor antigen–specific CD8⁺ T cell responses [303, 304]. Illustrating this concept, SNAPvax™, a novel nanoparticle platform co-delivering peptide antigens and TLR agonists, has shown significant promise in combination with oncolytic HSV. This combination improved survival, reduced tumor volume, and enhanced intratumoral viral replication and CD8⁺ T cell infiltration in murine models [305]. Similarly, Atherton et al. reported on the efficacy of a synthetic long peptide (SLP) vaccine targeting HPV16/18 antigens combined with MG1-E6E7, an oncolytic Maraba virus, for the treatment of HPV-associated malignancies. In mice bearing established tumors, the combination resulted in complete tumor eradication in 60% of animals and conferred durable protection against subsequent aggressive rechallenge, highlighting its potential for achieving long-term antitumor immunity [306].

### Nucleic acid vaccines: DNA and mRNA platforms with OV delivery

Nucleic acid vaccines, including those based on DNA and mRNA platforms, offer distinct advantages over protein-based vaccines by eliciting more comprehensive and durable immune responses through endogenous antigen production within host cells. Lopes et al. demonstrated the enhanced efficacy of a multiepitope plasmid DNA (pDNA) vaccine when combined with an oncolytic Adv in a melanoma model. The combined treatment markedly amplified immune activation within the TME, promoting robust NK cell infiltration and elevating the production of cytokines, chemokines, and immunomodulatory enzymes. Immunophenotyping analyses further revealed substantial increases in NK cells, CD4⁺ helper T cells, and CD8⁺ cytotoxic T cells in the combination group compared to monotherapy arms [307].

The success of mRNA vaccines in infectious diseases has accelerated their application in oncology, showcasing considerable promise for cancer immunotherapy [308–310]. Fu et al. developed a recombinant VSV engineered to minimize induction of neutralizing antibodies (rVSV-LCMVG) and combined it with an mRNA vaccine to enhance antitumor immunity against solid tumors. While intratumoral administration of either rVSV-LCMVG or mRNA vaccine alone moderately suppressed tumor growth, their combination, whether delivered intratumorally or intravenously, significantly enhanced antitumor efficacy and extended survival in murine models. Intravenous co-administration proved more effective, likely due to the induction of stronger systemic immune responses and expansion of antigen-specific T cell populations [311].

In a complementary strategy, Zhang et al. engineered an HSV-1–based OV, T22H07, to express HPV immunogens, and paired it with a therapeutic mRNA vaccine, M22H04, targeting HPV-associated malignancies. This regimen exemplifies a prime–boost vaccination strategy in the context of OV-based immunotherapy, in which priming with M22H04 initiates antigen-specific T cell responses in the periphery, followed by intratumoral boosting with T22H07. The OV not only delivers the same tumor-associated antigens but also remodels the TME through oncolysis and local inflammation. Together, this sequential approach enhances both systemic and intratumoral immunity, promoting immune cell infiltration—particularly cytotoxic CD8⁺ T cells—and driving myeloid and lymphoid compartments toward antitumor phenotypes [312].

In OV-based combination strategies, protein/peptide and nucleic acid vaccines exhibit distinct yet complementary immunological profiles. Protein and peptide vaccines are characterized by favorable safety and stability but rely on exogenous antigen uptake and therefore benefit primarily from OV-mediated enhancement of antigen presentation and local inflammation. In contrast, nucleic acid vaccines enable endogenous antigen expression, promoting efficient MHC class I presentation and robust CD8⁺ T cell priming, which aligns closely with OV-driven intracellular antigen processing pathways. In addition, the encoding flexibility of nucleic acid platforms facilitates multivalent and personalized antigen design. Collectively, both modalities are compatible with OV-based therapies, with their optimal application determined by antigen format and therapeutic context.

## Harnessing small-molecule immunomodulators to augment oncolytic virotherapy

Small-molecule immunomodulators represent a pharmacologically diverse class of low-molecular-weight compounds capable of precisely regulating immune cell functions, modulating key signaling pathways, and controlling the release of inflammatory cytokines to either activate or suppress immune responses [313, 314]. These compounds encompass a broad spectrum of agents, including naturally derived bioactive substances [315] and synthetically designed molecularly targeted drugs [316]. Their mechanisms of action are multifaceted and context-dependent, ultimately aiming to enhance the host’s innate and adaptive defense mechanisms, restore immune homeostasis, and effectively combat a range of pathological conditions, including infectious diseases, autoimmune disorders, and malignancies.

### Natural products as immune-potentiating agents for oncolytic virotherapy

Naturally derived bioactive compounds constitute an important class of small-molecule immunomodulators widely sourced from plants, animals, and microorganisms (Table 6) [317–319]. These substances exhibit diverse immunostimulatory properties: polysaccharides such as those from Ganoderma lucidum and Lycium barbarum activate macrophages and T cells, promote cytokine secretion, and enhance nonspecific immune responses [320, 321]. Similarly, active components in botanical extracts like Astragalus membranaceus [322] and Panax ginseng [323] have been shown to modulate immune cell functions, exert antioxidative and anti-inflammatory effects, and bolster overall immune competence [324].

**Table 6:** Small-molecule immunomodulators combined with oncolytic viruses

Category	Representative agents	Mechanism of action (immunomodulatory)	Representative OVs	Tumor models
Natural products	Cytokine [326], curcumin [325, 359, 360], resveratrol [361], triptolide [362], luteolin [328], olive leaf extract [329], ursolic acid [363], melittin [331], Lactobacillus casei extract [332]	Modulate NF-κB/STAT3/ROS → reduce immunosuppression, enhance ICD and antitumor immunity	VSV [325, 361, 362], HSV [359], Adv [326, 360], VV [328], NDV [329, 332], MV [363], coxsackieviruses [331]	Prostate cancer, melanoma, colorectal cancer, breast cancer, hepatocellular carcinoma
HDAC inhibitors	Vorinostat [364], Panobinostat [365], Valproic acid [366], trichostatin A [367, 368]	Inhibit histone deacetylases → induce chromatin relaxation and transcriptional reprogramming; enhance tumor antigen presentation (↑MHC-I), upregulate co-stimulatory molecules, and modulate cytokine expression	HSV[365, 366], VV[368], Adv [364, 367]	Glioma, squamous cell carcinoma, melanoma, colorectal, esophageal squamous cell carcinoma
mTOR inhibitors	Rapamycin [344, 369], Everolimus [370]	Inhibit mTOR signaling → regulate T cell differentiation and metabolism; suppress effector T cell overactivation while promoting memory T cell formation	HSV [344], Adv [370], Myxoma Virus [369]	Colon Cancer
MAPK pathway inhibitors	Vemurafenib [345], Trametinib [371]	Inhibit MAPK signaling → reduce tumor cell proliferation; modulate tumor antigen expression and improve immune recognition	HSV [345, 371]	Melanoma, brain tumors
JAK/STAT pathway inhibitors	Ruxolitinib [341]	Block cytokine signaling pathways → regulate inflammatory responses and immune cell activation	VSV [341]	Ovarian cancer

OVs, oncolytic viruses; VSV, vesicular stomatitis virus; HSV, herpes simplex virus; Adv, adenovirus; VV, vaccinia virus; NDV, Newcastle disease virus; MV, measles virus; NF-κB, nuclear factor kappa B; STAT3, signal transducer and activator of transcription 3; ROS, reactive oxygen species; ICD, immunogenic cell death; mTOR, mechanistic target of rapamycin; MAPK, mitogen-activated protein kinase; JAK, Janus kinase; STAT, signal transducer and activator of transcription; MHC-I, major histocompatibility complex class I

The combination of natural products with OVs represents an innovative antitumor strategy that leverages dual mechanisms to enhance therapeutic outcomes [325] (Fig. 5). While OVs are frequently engineered to express cytokine genes, alternative approaches include the direct co-administration of cytokines with OVs. For example, Wang et al. demonstrated that combining IFNα with the oncolytic Adv SG600-IL-24 yielded promising results in preclinical models of hepatocellular carcinoma (HCC). This regimen significantly suppressed HCC cell proliferation and migration, induced apoptosis, and inhibited metastasis and angiogenesis via modulation of the STAT1/SOCS1/STAT3 signaling axis and downstream effector proteins [326].

Owing to their favorable safety profiles and pleiotropic bioactivities, natural products continue to serve as valuable sources for antitumor drug discovery [327]. Wang and colleagues combined luteolin, a plant-derived flavonoid known to enhance macrophage phagocytosis and immune activation, with an oncolytic VV (VV-IL-24), observing superior tumor growth inhibition compared to either monotherapy [328]. Golalipour et al. reported that olive leaf extract encapsulated in lipid nanoparticles synergized with NDV to exert potent cytotoxicity against cervical cancer cells in vitro [329]. Likewise, Liu et al. found that ursolic acid significantly augmented the oncolytic activity of MV against breast cancer cells by enhancing apoptosis, suggesting a novel combinatorial strategy for breast cancer treatment [330].

Recent innovative approaches include the use of engineered OVs with natural immune complexes. Bahreyni et al. developed a combination therapy using miR-CVB3, a genetically modified OV, along with a melittin/CpG oligonucleotide complex (CpGMel) [331]. The OV mediates tumor cell lysis and antigen release, while melittin facilitates CpG uptake via membrane permeabilization, activating TLR9 signaling and promoting DC maturation. This combination induced robust DAMPs release, enhanced infiltration of CD8⁺ T and NK cells, and elevated IFN-γ expression, leading to reduced primary tumor burden and suppression of pulmonary metastases in vivo [331]. In another study, Ghorbani Alvanegh et al. demonstrated that NDV delivered via mesenchymal stem cells combined with Lactobacillus casei extract elicited significant antitumor effects in colorectal cancer models, increasing lactate dehydrogenase release and reactive oxygen species (ROS) production and promoting apoptotic cell death [332].

In summary, the integration of natural products with oncolytic virotherapy not only broadens the arsenal of cancer treatment options but also underscores a shift toward multimodal, multi-target therapeutic strategies aimed at achieving synergistic antitumor efficacy.

### Molecularly targeted drugs for pathway-specific synergy with OVs

While natural active substances modulate the immune microenvironment through multi-pathway regulation, molecularly targeted drugs provide a rationally designed, mechanism-based strategy to enhance oncolytic virotherapy. Molecularly targeted drugs are increasingly recognized as a novel class of small-molecule immunomodulators with precise mechanisms of action [333]. These compounds specifically bind to and modulate key immune-related molecules or signaling pathways, such as TLRs [334] and cytokine receptors [335], to influence immune cell activation, proliferation, differentiation, and functional polarization [336]. Compared to conventional therapeutics, molecularly targeted drugs exhibit superior selectivity and reduced off-target effects, allowing for more precise immune regulation. They hold particular promise for treating complex diseases including autoimmune disorders, chronic inflammation, and cancer immune evasion [337, 338].

The combination of molecularly targeted drugs with OVs offers a synergistic approach that merges precise molecular intervention with robust immune activation, significantly improving antitumor efficacy. For instance, Janus kinase (JAK) inhibitors disrupt cytokine signaling by inhibiting JAK enzymatic activity, thereby modulating immune cell proliferation, differentiation, and inflammatory responses [339, 340]. Research by Geoffroy et al. demonstrated that combining oncolytic VSV with JAK inhibitors, such as ruxolitinib, baricitinib, and fedratinib, attenuated tumor cell antiviral defenses by blocking IFN signaling pathways, thereby augmenting VSV-mediated oncolysis. These findings underscore the therapeutic potential of combining VSV with JAK inhibitors for ovarian cancer treatment [341].

Nakatake et al. further explored the utility of histone deacetylase inhibitors (HDACis) in enhancing the efficacy of oncolytic VV (FUVAC). HDACis suppressed host antiviral defense mechanisms, promoted viral replication, and amplified systemic antitumor immunity by facilitating enhanced cell–cell fusion [342]. Similarly, Miao et al. reported that bromodomain-containing protein 4 (BRD4) inhibitors significantly boosted the replication and therapeutic potency of oncolytic Adv HAdVC5 in pancreatic ductal adenocarcinoma models. BRD4 inhibition increased viral E1A expression, altered cell cycle regulators and inflammatory mediators, and downregulated oncogenes such as c-Myc and Myb, collectively enhancing oncolytic efficacy [343]. In addition, rapamycin, an mTOR inhibitor, has been reported to enhance the yield and intratumoral dissemination of oncolytic HSVs in semipermissive tumor cells, thereby overcoming intrinsic resistance to viral replication and significantly improving antitumor efficacy in vivo [344].

Modulation of oncogenic signaling pathways can further potentiate OV-induced antitumor immunity. MEK inhibition has been shown to enhance OV efficacy by promoting CD8⁺ T cell activation, increasing dendritic cell–mediated antigen presentation and antigen spreading, and inducing a more inflamed TME; concomitant upregulation of PD-L1 also provides a rationale for combination with immune checkpoint blockade, with triple therapy demonstrating superior therapeutic outcomes [345]. Yamada et al. proposed an innovative multi-target strategy combining focal adhesion kinase inhibitors (FAKis) with the oHSV G47Δ and immune checkpoint blockers for pancreatic cancer treatment. This approach combines direct oncolysis with immunomodulation: FAKis inhibit stromal support for tumors, recruit CTLs, and reduce regulatory T cell (Treg) infiltration, thereby remodeling the TME. When combined with G47Δ, which directly lyses tumor cells and releases TAA, the treatment promotes potent and systemic antitumor immunity [346].

The combination of OVs with small-molecule immunomodulators, encompassing both natural products and molecularly targeted drugs, provides a powerful strategy to enhance antitumor efficacy through precise pharmacological manipulation of the TME and immune signaling. Natural bioactive compounds, such as plant polysaccharides and flavonoids, augment oncolytic virotherapy by promoting immune cell activation and cytokine release. Concurrently, targeted agents including JAK inhibitors, HDAC inhibitors, and BRD4 or FAK blockers synergize with OVs by suppressing antiviral defenses, enhancing viral replication, and reprogramming immunosuppressive pathways. Meanwhile, early-phase clinical investigations have begun to explore OV-based combinations with small-molecule agents, including kinase inhibitors, JAK/STAT pathway inhibitors, and immunomodulatory chemotherapeutics such as cyclophosphamide. These studies primarily aim to modulate antiviral responses and improve viral persistence or tumor susceptibility, although the number of such trials remains limited and most are still in exploratory stages. Together, these approaches enable multifaceted immune activation and stromal remodeling, significantly improving systemic antitumor responses and offering a refined combinatorial platform for next-generation viro-immunotherapy.

## Conclusions and prospects

The expanding application of OVs in combination with diverse immunotherapeutic modalities highlights their considerable promise in modern cancer treatment. As multifunctional immunotherapeutic agents, OVs not only selectively lyse tumor cells but also induce immunogenic cell death and reshape the TME, thereby enhancing antitumor immunity. Their integration with immune checkpoint inhibitors, cell therapies, cancer vaccines, and small-molecule immunomodulators reflects a shift toward more personalized and synergistic therapeutic strategies. In addition, advances in genetic engineering have enabled the development of OVs with improved tumor selectivity, enhanced safety, and optimized transgene delivery, further strengthening their capacity to induce durable immune responses.

Despite these advances, several challenges continue to hinder the clinical translation of OV-based therapies. Manufacturing complexity, high production costs, and stringent storage requirements limit large-scale application. Meanwhile, delivery remains a major obstacle: intratumoral injection is restricted to accessible lesions, whereas systemic administration is often compromised by rapid immune clearance and off-target sequestration. Furthermore, intrinsic antiviral responses and tumor heterogeneity can impair viral replication and intratumoral spread. Although OV monotherapy is generally well tolerated, it may still cause flu-like symptoms and, in rare cases, organ-specific toxicities. Notably, combination strategies, while enhancing efficacy, may also increase the risk of immune-related adverse events due to excessive immune activation. In addition, antiviral immunity may paradoxically limit viral persistence, underscoring the need to balance immune activation with viral fitness.

To address these limitations, several strategic directions are emerging. Rational combination approaches targeting key immunosuppressive pathways may help overcome resistance and improve therapeutic efficacy. Modulation of the microbiota–immune axis and reprogramming of immune cell metabolism represent promising avenues to further enhance antitumor responses. In parallel, the development of next-generation OVs incorporating advanced payloads, such as cytokines, non-coding RNAs, and genome-editing tools, may improve precision and functionality. Importantly, the integration of multiomics data and artificial intelligence is expected to refine patient stratification and guide personalized treatment design, thereby maximizing therapeutic benefit.

In conclusion, OVs represent a dynamic and evolving platform in cancer immunotherapy. Their unique ability to synergize with multiple therapeutic modalities, combined with their versatility as delivery vehicles, positions them as key components of next-generation combination strategies, with the potential to achieve more durable and effective antitumor responses.

## Data Availability

No datasets were generated or analysed during the current study.

## Abbreviations

OVs: Oncolytic viruses
TME: Tumor microenvironment
ICIs: Immune checkpoint inhibitors
Adv: Adenoviruses
VV: Vaccinia virus
HSV: Herpes simplex virus
RV: Reovirus
MV: Measles virus
NDV: Newcastle disease virus
VSV: Vesicular stomatitis virus
T-VEC: Talimogene Laherparepvec
ICD: Immunogenic cell death
AI: Artificial intelligence
TAAs: Tumor-associated antigens
ICP: Infected cell protein
GM-CSF: Granulocyte-macrophage colony-stimulating factor
DCs: Dendritic cells
Tregs: Regulatory T cells
MDSCs: Myeloid-derived suppressor cells
MSE: Mean squared error
PD-1: Programmed death receptor 1
ORR: Objective response rate
NE: Neutrophil elastase
PPE: Porcine pancreatic elastase
TAM: Tumor-associated macrophage
αGal: α-galactosidase
ADCC: Antibody-dependent cellular cytotoxicity
IFN: Interferon
TNF-α: Tumor necrosis factor-α
TRAIL: Tumor necrosis factor-related apoptosis-inducing ligand
CTLA-4: Cytotoxic T lymphocyte-associated antigen-4
IL: Interleukin
Tα1: Thymosin α1
TAMs: Tumor-associated macrophages
TIGIT: T-cell immunoglobulin and the ITIM domain
NK: Natural killer
TIM-3: T-cell immunoglobulin and mucin domain-3
OS: Overall survival
iRFA: incomplete radiofrequency ablation
TIL: Tumor-infiltrating lymphocyte
MYXV: Myxoma virus
CAR: Chimeric antigen receptor
TCR: T-cell receptor-engineered
CIK: Cytokine-induced killer
CXCR: C-X-C chemokine receptor type
CXCL: Chemokine (C-X-C motif) ligand
MHC: Major histocompatibility complex
FDA: Food and drug administration
TCRs: T-cell receptors
EwS: Ewing sarcoma
aAPCs: artificial antigen-presenting cells
PBMCs: Peripheral blood mononuclear cells
SIRP: αSignal regulatory proteinα
HPV: Human papillomavirus
PAMPs: Pathogen-associated molecular patterns
DAMPs: Damage-associated molecular patterns
SLP: Synthetic long peptide
HCC: Hepatocellular carcinoma
STAT: Signal transducer and activator of transcription
TLRs: Toll-like receptors
JAK: Janus kinase
HDACis: Histone deacetylase inhibitors
BRD4: Bromodomain-containing protein 4
FAKis: Focal adhesion kinase inhibitors
OX40: Oxford 40
ROS: Reactive oxygen species
